# HIV self-testing preferences among adolescent girls and young women (AGYW) in urban settings in Uganda: A discrete choice experiment

**DOI:** 10.1371/journal.pgph.0005431

**Published:** 2026-09-22

**Authors:** Laban Muteebwa, Patience A. Muwanguzi, Shivan Nuwasiima, Ivan Ahimbisibwe, Edson Atwine, Dan Muramuzi, Cathbert Tumusiime, Fred C. Semitala, Joanita Nangendo

**Affiliations:** 1 Department of Medicine, School of Medicine, College of Health Sciences, Makerere University, Kampala, Uganda; 2 Department of Nursing, School of Health Sciences, College of Health Sciences, Makerere University, Kampala, Uganda; 3 Clinical Epidemiology Unit, School of Medicine, College of health Sciences, Makerere University, Kampala, Uganda; 4 Faculty of Graduate Studies, Makerere University Business School, Makerere University, Kampala, Uganda; 5 Department of Pharmacy, School of Health Sciences, College of Health Sciences, Makerere University, Kampala, Uganda; 6 Department of Health Policy Planning and Management, School of Public Health, College of Health Sciences, Makerere University, Kampala, Uganda; 7 Directorate of Research, Baylor College of Medicine Children’s Foundation-Uganda, Kampala, Uganda; 8 Department of Programs, Makerere University Joint AIDS Program, Kampala, Uganda; 9 Research Department Infectious Disease Research Collaboration, Kampala, Uganda; University of California San Francisco, UNITED STATES OF AMERICA

## Abstract

HIV self-testing (HIVST) is a crucial innovation for improving testing coverage, particularly among adolescent girls and young women (AGYW) who are inadequately reached by standard provider-assisted HIV testing approaches. Understanding their preferences for HIVST services is essential for designing models that optimize uptake. We assessed the HIVST preferences among AGYW at a higher risk of acquiring HIV living in urban Uganda. We enrolled AGYW at a higher risk of HIV using a community-based convenience sampling in a discrete choice experiment (DCE). Each participant completed eight choice sets, each comprising of two hypothetical HIVST service alternatives and an opt-out option. The four attributes included level of assistance (assisted versus unassisted), distribution model (facility-based versus community-based), delivery channel (primary versus secondary) and sample type (oral swab versus blood). A mixed logit model was used to estimate the marginal utilities, relative importance (RI), HIVST profile utilities and predicted uptake (compared to opt-out). Between December 2024 and May 2025, 340 participants were enrolled with mean age 19.8 years (standard deviation (SD): 2.6), and 65.9% of them were not in school. In the main effects model, participants preferred; facility-based over community-based distribution model (β = 0.14 (95% confidence interval (CI): 0.06, 0.23)), primary over secondary delivery channel (β = 0.09 (95% CI: 0.00, 0.17)), and unassisted over assisted testing (β = 0.46 (95% CI: 0.37, 0.54). The sample type didn’t significantly influence the preferences of participants to use HIVST (β = 0.05 (95%CI: -0.04, 0.13). The level of assistance emerged as the most important attribute (RI: 60%), followed by facility-based distribution model (RI: 18.9%) and primary delivery channel (RI: 12.2%). There was a significant interaction between delivery channel and level of assistance (β = 0.66 (95% CI: 0.07, 1.24)). Primary delivery was preferred over secondary delivery when testing was unassisted (β = 0.41 (95% CI: 0.11, 0.71), but not when testing was assisted (β = -0.25 (95% CI: -0.56, 0.06). AGYW preferred HIVST models combining primary delivery with unassisted testing while facility-based distribution also increased preference. This highlights the importance of considering both individual attributes and their interaction when designing HIV delivery models.

## Background

Global HIV infections have declined by 40% since 2010 – from 2.2 million to 1.3 million in 2024, however, progress towards combating new infections remains inadequate [1]. The continued shortfall from the 2025 target of fewer than 370,000 new infections, highlights persistent weaknesses in prevention and control efforts [1]. In Sub-Saharan Africa (SSA), women and girls continue to bear a disproportionate HIV burden, accounting for 63% of new infections in 2024 [1]. Within this group, adolescent girls and young women (AGYW) aged 15 – 24 years are the most severely affected where in 2024 alone, more than 4,000 AGYW were infected with HIV every week globally, of which over 82.5% occur in SSA [1]. In Uganda, the gendered impact is even more pronounced. In 2024, approximately 80% of all new HIV infections among young people aged 15 – 24 years occurred in AGYW [2]. A recent analysis of HIV testing data (November 2019 – November 2021) further revealed that, among 2,672 new infections in AGYW, about 87% had a long-standing undiagnosed HIV infection (more than 12 months since HIV acquisition) [3]. Furthermore, one in three AGYW with recent HIV infection were first-time testers [3], underscoring the critical need to expand routine and accessible HIV testing, particularly for AGYW at a higher risk of acquiring HIV.

HIV testing is the gateway to HIV prevention, care, and treatment, and constitutes the first pillar of the UNAIDS 95-95-95 target which aims to get 95% of people living with HIV to know their status, 95% of those diagnosed to receive treatment, and 95% of those on treatment to achieve viral suppression [4]. In Uganda, the Ministry of Health (MoH) has expanded provider-assisted HIV testing through various approaches, including facility-based (provider-initiated and client-initiated), community outreach, home-based and index testing [5]. Despite these efforts, conventional strategies have not optimally reached some sub-populations, particularly AGYW, female sex workers, men, and men who have sex with men (MSM) [6]. Among AGYW, low HIV testing uptake has been attributed to developmental factors intertwined with interpersonal, cultural, structural, and health system barriers, including stigma, limited privacy, long waiting times, negative provider attitudes and inadequate youth-friendly services [7]. HIV self-testing (HIVST) offers a promising convenient, confidential, and youth-responsive alternative to address these challenges [8–10].

HIVST is a “test-for-triage” approach in which individuals collect their own specimen (oral fluid or blood), perform the test, and interpret the results independently or with a trusted person [11]. Evidence from randomized controlled trials and implementation studies demonstrates that HIVST significantly increases testing uptake compared with the conventional provider-assisted testing approaches and is highly acceptable across diverse populations, including AGYW [12–14]. In Uganda, HIVST was incorporated into national HIV guidelines in 2018 [6], and is being scaled up in public and private sectors through various differentiated service delivery (DSD) models [5]. However, the heterogeneity of DSD models complicates efforts to tailor HIVST services to priority sub-populations such as AGYW in a manner that optimizes service uptake at minimal implementation costs [15,16]. Emerging evidence from stated preference studies show that HIV testing decisions are shaped by not only the service availability, but also how services are delivered [17]. For example, among young people in South Africa, HIV testing preferences were influenced by testing modality, social support and service location, thus demonstrating that youth value privacy and accessible service models [18]. While in Nigeria, young people preferred HIV testing services that were free, offered in hospitals or at home, and linked to treatment access [19]. In this study, young people preferred HIVST kits delivered through community health centers, free of charge, approved by World Health Organization (WHO) and accompanied by post-test support options [19]. In Uganda, though young women have shown high willingness to use HIVST [14,20], evidence about the preferences for specific service delivery attributes is limited. Understanding HIVST service delivery attributes that are most valued by AGYW is therefore critical for designing AGYW-centered, cost-efficient models that optimize uptake. Prioritizing delivery models aligned with AGYW preferences has potential to enhance testing coverage and HIV positivity yield while improving resource efficiency. This study aimed to assess HIVST service preferences and key attributes associated with improved testing uptake among AGYW in urban Uganda.

## Methods

### Ethics statement

The study was approved by the Makerere University School of Medicine Research Ethics Committee (Reference: Mak-SOMREC-2023–843), and further clearance was obtained from the Uganda National Council for Science and Technology (UNCST) (Reference: HS5167ES). Administrative approvals to conduct the study were secured from the Kampala Capital City Authority (KCCA) and the Municipal Health Offices of Entebbe and Nansana in Wakiso District. Written informed consent was obtained from all adult participants and emancipated minors. For other participants below 18 years, written informed assent was obtained alongside written informed consent from their guardians, in accordance with UNCST guidelines [21].

### Discrete choice experiments

This study utilized a discrete choice experiment (DCE), grounded in Lancaster’s theory of consumer demand [22], and the Random Utility Theory (RUT) [23] that underpins modelling of choice data. Under the RUT, when an individual is presented with two or more alternatives, they will choose an alternative that maximizes utility (benefit). This latent utility is a function of; (1) a systematic (observable) component which is determined by the measurable attributes of the alternatives and the characteristics of the individual; and (2) a random (unobserved) component which captures the factors not included in the model or randomness in decision making. The utility (U_ij_) for an individual (i) choosing alternative (j) is expressed as; $U_{ij}=\;V_{ij}+\;\varepsilon_{ij}$**,** Where; V_ij_ is the observable (deterministic) component –a linear combination of attributes, and Ɛ_ij_ is the unobserved (random) component. DCEs provide a structured and evidence-based method for eliciting stated preferences about health service delivery attributes [24]. By presenting participants with hypothetical service options composed of varying features (attributes), DCEs allow researchers to estimate the relative importance of each attribute, simulate uptake under different scenarios and identify trade-offs that end-users are willing to make [25].

### Study design and setting

This cross-sectional study was conducted among AGYW living in the Kampala Metropolitan Area in Uganda from 1^st^ December 2024–31^st^ May 2025. This study setting was selected because prior assessment in Sub-Saharan Africa reported that adolescents and young people living in urban settings have a 1.5 to 2 times HIV prevalence compared to their counterparts in rural settings [26]. Kampala Metropolitan Area includes the four most populous districts in Central Uganda, that is, Kampala, Wakiso, Mukono and Mpigi according to the 2024 National census [27]. This is the most urbanized and commercial area in Uganda and includes its Capital City, Kampala, hosting an estimated daytime population of about five million people [28]. Moreover, this study setting accounted for the largest number of new HIV cases of which Wakiso had the highest (4,900) followed by Kampala (2,800), Mukono (700) and Mpigi (405) [4]. Additionally, the HIV prevalence among adults aged 15–49 years (2023) in Kampala, Wakiso, Mpigi and Mukono is 7.4%, 7.2%, 8.2% and 5.3% respectively, all above the national average (2023) of 5.1% [4].

### Study population

The target population for this study was AGYW aged 15–24 years living in urban communities. Eligibility screening was conducted prior to enrolment where individuals were eligible if they: (1) were born female; (2) age 15–24 years; (3) had lived in the study area for at least six months; (4) were able to communicate in English or Luganda (a commonly spoken local language in the study setting); (5) had an HIV risk score ≥2, indicating high risk of HIV acquisition based on HIV risk assessment tool (S1 File) used by the Uganda Ministry of Health [5] and previously used in Kampala among AGYW [29]; and (6) gave written informed consent or assent in addition to guardian’s consent for minors. The HIV risk assessment tool had seven binary questions (yes/no) assessing the HIV risk behaviors. It asked about having had sexual intercourse, more than one sexual partner, consistent condom use, sexual intercourse with known person living with HIV, sexually transmitted infection (STI) or abnormal vaginal discharge and sharing injections, all in the past six months, and history of using post exposure prophylaxis for more than three times in the past 12 months. An individual was categorized as being at a high risk of acquiring HIV if they had a score of ≥2 of which “1” is for being sexually active (at least one episode of sexual intercourse in past six months). Following this initial eligibility screening, participants were assessed for willingness to use HIVST service if it were provided, by using the Theoretical Framework of Acceptability (TFA) as previously reported [14]. This framework has seven domains for which each domain was assessed using a one 5-level Likert item question weighted 1 – 5 for which a higher score indicated a higher level of acceptability. The sum of weights from all seven domains was computed and a score above the 50^th^ percentile of the possible scores (which is 21) ranging from 7 – 35 was used as a threshold of willingness to use HIVST. The participants who had a willingness score above 21 (out of maximum score of 35) were deemed to be willing to use HIVST and were included in the DCE. Individuals who did not meet the eligibility criteria at screening were excluded prior to enrolment, while those who met the screening criteria but did not meet the willingness to use HIVST threshold were also excluded from the DCE analysis.

### Sample size estimation and sampling procedure

The sample size was estimated using the Orme and Johnson formula [30] $N>\frac{\text{5}\text{0}\text{0}*c}{t*a}$ where, “N” is the estimated sample size, “c” is the maximum number of levels per each attribute, “t” is the number of tasks and “a” is the number of alternatives per task excluding the null alternative (opt-out). In this study, “c” was 2, “t” was 8 and “a” was 2. Adjusting for 10% non-response and design effect (due to use of multi-stage sampling design) of two as recommended by WHO [31], the estimated sample size was 140. However, this DCE study was nested in a larger study that assessed the willingness to use HIVST among AGYW [14]. All participants that demonstrated willingness to use HIVST (score >21) were enrolled into the DCE study, thus a total 340 AGYW were included.

The sampling was done on three levels – firstly, two (Kampala and Wakiso) out of four districts were selected purposively because they had the highest number of new HIV infections among AGYW. Secondly, two (Makindye and Rubaga) out of five divisions in Kampala district and two (Entebbe and Nansana) out of four municipalities in Wakiso district were selected using simple random sampling. Lastly, the study participants were selected using convenience sampling technique – where all eligible individuals were screened and enrolled on a rolling basis until the desires sample size was achieved. Participant recruitment was conducted through targeted community-based mobilization in selected urban areas within Kampala and Wakiso districts. These areas included locations identified as having higher concentrations of AGYW that are at an elevated risk of HIV acquisition such as; informal settlements, trading areas and social venues (like bars, clubs, discotheques, cinema halls and taverns). Trained research assistants, with the support of community health workers (also known as Village Health Teams in Uganda) and peer mobilizers approached potential participants within these community settings and provided information about the study. Screening for eligibility was conducted in the community and all AGYW who met the inclusion criteria during the recruitment period were invited to participate. The number of participants recruited from each district and division/municipality was proportionately allocated based on the 2023 projected number of AGYW [32].

### Experimental design for a DCE

The attributes of HIVST service were derived from the MoH consolidated guidelines for prevention and treatment of HIV (2022) [5] to conform with the existing DSD models that are currently being implemented for HIVST. However, the distribution model of the HIVST services delivery through the private sector was not considered in this study. Therefore, only four attributes of the HIVST service were considered, of which all had two levels. These include; distribution model (Facility-based versus community-based), delivery channel (primary delivery source versus secondary delivery source), sample/kit type (oral swab versus blood), and level of assistance (assisted versus unassisted) (Table 1). With the four attributes of the HIVST services considered, each with two levels, a full factorial experimental design would give rise to 16 choice sets (2^4^). However, only eight choice sets (fractional factorial) were considered as it has been reported to be optimal to elicit the desired utility measures while preventing cognitive overload of study participants [33,34]. To select the choice sets to be considered, the D-efficient design was implemented in STATA version 17.0 (Texas USA) with the assumption that no prior coefficient estimates of the attributes in the target population exist (no priors’ matrix), including the alternative specific constant. Given attribute level matrix of 2, 2, 2, 2 (each has two levels) and two alternatives plus the opt-out (opt-out matrix of 1, 1, 1, 1), the modified Fedorov algorithm [35–37] – which maximizes the D-efficiency of the design based on covariance matrix of a conditional logit model, was used to generate eight choice sets. The generated choice sets were used to design the DCE questionnaire (S2 File).

**Table 1:** HIVST service attributes and levels adapted from the Consolidated guidelines for prevention and treatment of HIV in Uganda.

Attribute and definition	Level	Attribute level definition
Distribution Model (Refers to where the HIVST services are offered and accessed from)	Ⅰ. Facility-based	Refers to where HIVST services are delivered to end-users or their social contacts at designated health facilities. The HIVST services are given at various departments of both public and private health facilities including pharmacies and drug shops. The health facility departments may include Out-Patient Department (OPD), In-patient Department, Maternal and Child Health clinic, ART clinics, Family Planning/Sexually Transmitted Infections clinics and adolescent/youth corners.
	Ⅱ. Community-based	Community-based model is where HIVST services are provided to users or their social contacts in the communities themselves. The HIVST services are decentralized from formal health facilities to the community. It includes community-based organizations, work places, drop-in centers, house-to-house distribution and mobile distribution
Delivery channel (Refers to how HIVST kits are provided to an individual who needs HIVST service)	Ⅰ.Primary	This is where HIVST kits are delivered directly to the end user (end user gets directly from the distribution point)
	Ⅱ. Secondary	This is where HIVST kits are delivered to the end user indirectly through intermediaries who include a friend, partner, peer leader, social contact, village health team (VHT) or community health worker (CHW)
Sample type/test kit type (Refers to the type of specimen sample that must be collected to use an HIVST kit)	Ⅰ. Oral swab	Oral swab that is used by an HIV self-test kit designed to use oral fluid sample
	Ⅱ. Blood	Blood sample that is used by an HIV self-test kit that is designed to use a blood sample collected by a simple finger prick
Level of assistance (Refers to the level of assistance available to support a client in administering the HIVST and interpreting of the test result)	Ⅰ.Assisted self-testing	“Assisted” is where the instructions and support are provided by trained personnel that a client is comfortable with
	Ⅱ. Unassisted self-testing	“Unassisted” is where the client/ end-user administers the self-test and interprets the test result independently (without any assistance)

### Data collection tool and procedure

**Fig 1:**
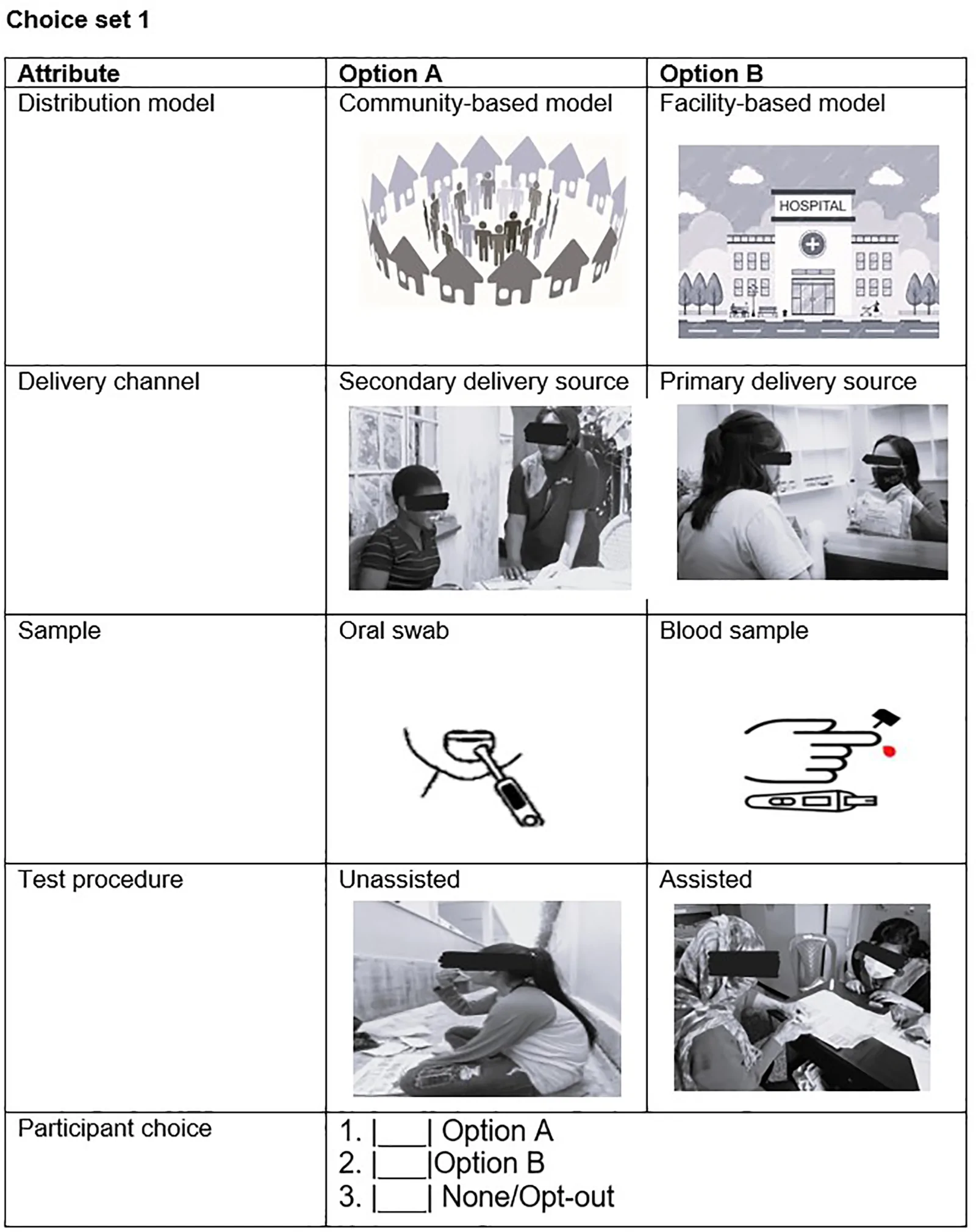
An example choice scenario presented to the study participants.

A structured questionnaire was used to collect the data on the socio-demographic, and behavioral characteristics, as well as the discrete choices that describe the DSD models for HIVST service. The questionnaire had a detailed explanation of the HIVST service attributes which were first explained to each participant before engaging in selection of preferred service profiles. The choice sets of the HIVST service attributes and levels assessed in this study are attached as S2 File. The DCE questionnaire was further enhanced with images describing the attributes of HIVST services which were obtained from a public database of images (noun project) [38]. An example of a choice scenario is presented in Fig 1. The questionnaire was pretested among 20 AGYW in Kawempe division in Kampala city, and these participants were excluded in this analysis. A “think-aloud” approach was used in the pretest to assess the participants’ understanding and thought process as they made choices about the tasks presented, and feedback was used to refine the questionnaire.

Targeted mobilization was carried out in the study areas to identify AGYW who are at an elevated risk of acquiring HIV. The information about HIVST was given and practical demonstrations of how the HIVST kits are used were done before screening the study participants. Both the oral (OraQuick, OraSure Technologies, Inc. Bethlehem, PA, USA) and blood-based (GOLDEN TIME, Gaobeidian PRISES Biotechnology Co., Ltd, Hebei Province, China) HIVST kits were used in the demonstrations. Trained research assistants administered the questionnaire to AGYW who were eligible for the study.

### Study outcomes

The primary outcome was the choice of preferred alternative of the tasks that offered the highest utility (benefit) to individual participants. The marginal utility gained from each of the attributes was computed as the primary measure. The Relative Importance (RI) was defined as the measure of influence each attribute had on participant’s decision to choose an alternative relative to other attributes in the model. Furthermore, the attribute tradeoffs – defined as the utility of one attribute level a participant is willing to give up (or accept less of) in order to gain a more preferred level of another attribute while still choosing the same alternative was also reported. The profile utility (the utility gained from combining the HIVST attributes) of the HIVST service models included was computed and ranked to show most preferred models. Furthermore, the predicted uptake of HIVST service when offered through the selected service profiles was also computed against the opt-out (not choosing the service profile).

### Statistical analysis

The data analysis was done in STATA version 17.0 (Texas, USA). The participant characteristics were summarized using descriptive statistics. Continuous variables were summarized using mean and standard deviation (SD) or median and interquartile range (IQR), while categorical variables were summarized using frequencies and percentages. The mixed logit model was used to fit a model with the outcome of choice, and attributes of HIVST services as the alternative-specific variables. Two discrete choice models were estimated; a main effects model and an interaction model (with significant interaction terms). While the interaction model provided a superior statistical fit, the main effects model was retained to derive overall population-level metrics including relative importance (RI) of attributes and preference trade-offs. The interaction model was subsequently utilized to examine how preferences systematically varied. The Relative Importance of an HIVST service attribute was computed as a proportion of the range of coefficients of an attribute out of the total sum of the ranges of coefficients of other attributes in the final model [39].


$$\begin{array}{c} \text{Relative importance }(\text{RI}k)\;=\;\frac{Max\left(\beta_{k}\right)-Min\;\left(\beta_{k}\right)}{\sum_{k=\text{1}}^{k}\left(Max\left(\beta_{k}\right)-Min\left(\beta_{k}\right)\right)} \end{array}$$

The Trade-offs between one attribute levels to another were computed as a ratio of coefficient of the preferred level to the sacrificed level [40].


$$\begin{array}{c} \text{Tradeoff }XY\;=\;\frac{\beta_{x}}{\beta_{Y}} \end{array}$$

We assessed two-way interactions between the distribution model, delivery channel and level of assistance by including the interaction terms in the model, and using manual stepwise approach. These interactions were considered due to their conceptual plausibility, and only statistically significant interaction terms were retained in the final model. The models were compared using the Akaike Information Criteria (AIC) and Bayesian Information Criteria (BIC), and the final model was used to compute the utilities of the 16 HIV service delivery profiles. The profiles were ranked starting with the most preferred HIVST delivery profile. We estimated the predicted uptake for each profile when offered against the opt-out (not choosing any profile). The opt-out option was retained in the simulation to estimate the proportion of participants who would decline HIVST service profile. As part of sensitivity analysis, we assessed straight-lining behavior as part of data quality checks – defined as consistently selecting the same alternative across all choice tasks, however, these participants were retained in the analysis, because as such behavior may reflect true strong preferences rather than inattentive responding. We assessed the internal validity using the leave-one-choice-set out sensitivity analysis. The mixed logit model was re-estimated after excluding one choice set at a time. Predicted choices for the omitted choice set were then compared with observed choices. Model stability was assessed by comparing the direction and magnitude of coefficients across reduced models.

## Results

A total of 377 participants were screened, of which 340 (90.2%) were willing to use HIVST and therefore enrolled in the stated preference elicitation exercise. All of the 340 participants were subjected to eight (8) choice sets (response rate 100%), with two alternative models and an opt-out option (choosing none of the alternatives presented). There was no missing data, and this gave rise to a total of 8,160 alternatives to choose from. Only 2.5% of the alternatives chosen were opt-out.

### Socio-demographic characteristics of study participants

The mean age of the participants was 19.8 years (SD: 2.6), about two-thirds (65.9%) were out of school and 71.8% didn’t live with their primary partner. 57.9% of the participants reported having no monthly income or did not answer, and among those with an income, the median monthly income was about United States Dollars (USD) $42.1 (IQR: 28.1, 56.1). Slightly more than half of participants (56.8%) had ever been pregnant and about a similar proportion (55.9%) were using a modern contraceptive method. Half of the participants (50.5%) had primary partners who were older by >5 years. 18.8% reported to have experienced intimate partner violence and 10.6% experienced sexual violence from their intimate partners in the past six months (Table 2).

**Table 2:** Socio-demographic characteristics of AGYW who participated in the study.

Variable	Category	Frequency (N = 340)	Percentage %)
Age (Years)	Mean (SD)	19.8 (2.6)	
Schooling status	In school	116	34.1
	Not in school	224	65.9
Live with partner	Yes	96	28.2
	No	244	71.8
Monthly income*	Median (IQR)	$42.1 (28.1, 56.1)	
Ever pregnant	Yes	193	56.8
	No	147	43.2
Contraceptive use in past six months	Yes	190	55.9
	No	150	44.1
Age difference with primary partner	≤5 Years	142	41.8
	>5 Years	198	58.2
Experienced intimate partner violence in past six months	Yes	64	18.8
	No	276	81.2
Experienced sexual violence in past six months	Yes	36	10.6
	No	304	89.4

*Median income for 143 participants (at USD 0.00028 = 1 Uganda shillings).

### Behavioral characteristics of the study participants

In the six months preceding the study, 55.6% of participants reported to have had a sexually transmitted infection (STI), 30.3% had multiple sexual partners, and 14.1% reported consistent condom use. Transactional sex was reported by 29.7%, anal sex by 11.2%, and intravenous drug use by 15.6%. Only 6.2% and 4.7% reported using Post Exposure Prophylaxis (PEP) and Pre-Exposure Prophylaxis (PrEP), respectively. Overall, 83.2% had ever tested for HIV, and 75.6% of these had done so within the past 12 months. While 71.2% had heard of HIV self-testing (HIVST), only 50.4% had ever used an HIVST kit, and 58.8% reported being unable to obtain one when they needed it. (Table 3).

**Table 3:** Behavioral characteristics of AGYW who participated in the study.

Variable	Category	Frequency (N = 340)	Percentage (%)
STI in past six months*	Yes	189	55.6
	No	151	44.4
Consistent condom use in past six months	Yes	48	14.1
	No	292	85.9
Had multiple sex partners in past six months	Yes	103	30.3
	No	237	69.7
Had anal sex in past six months	Yes	38	11.2
	No	302	88.8
Had sex for money/gifts in past six months	Yes	101	29.7
	No	239	70.3
Used injectable illicit drugs in past six months	Yes	53	15.6
	No	287	84.4
Used PEP in past six months^@^	Yes	21	6.2
	No	319	93.8
Used PrEP in past six months^#^	Yes	16	4.7
	No	324	95.3
Ever tested for HIV	Yes	283	83.2
	No	57	16.8
HIV test in past 12 months^$^	Yes	216	75.6
	No	69	24.4
Ever heard about HIVST	Yes	242	71.2
	No	98	28.8
Ever used HIVST^$$^	Yes	122	50.4
	No	120	49.6
Ever failed to get HIVST kit when in need of it	Yes	41	12.1
	No	99	29.1
	Never looked for it	200	58.8

*STI-Sexually Transmitted Diseases, ^@^PEP-Post-Exposure Prophylaxis, ^#^Pre-Exposure Prophylaxis, ^$^N = 285, ^$$^N = 242.

### Preference of the HIVST service attributes

The marginal utility estimates from the main effects mixed logit model showed that AGYW strongly preferred unassisted HIV self-testing compared to assisted testing (β = 0.46, 95% CI: 0.37, 0.54). Facility-based distribution was also preferred over community-based distribution (β = 0.14, 95% CI: 0.06, 0.23), as was primary delivery compared to secondary delivery (β = 0.09, 95% CI: 0.00, 0.17). In contrast, sample type had minimal influence on preferences, with no statistically significant difference between oral swab and blood-based testing (β = 0.05, 95% CI: −0.04, 0.13). Based on the estimates of the main effects model, the RI analysis showed that level of assistance was the most influential attribute in shaping HIV self-testing preferences (62.2%), followed by distribution model (18.9%) and delivery channel (12.2%), while sample type contributed the least to decision-making (6.8%) (Table 4).

**Table 4:** Main-effects model of HIVST service preferences of 340 AGYW who participated in the study.

Attribute	Attribute level	Coefficient (95% CI)	P value	Relative importance (%)
Distribution model	Facility-based	0.14 (0.06, 0.23)	0.001	18.9
	Community-based	Ref		
Delivery channel	Primary delivery	0.09 (0.00, 0.17)	0.042	12.2
	Secondary delivery	Ref		
Sample	Oral swab	0.05 (-0.04, 0.13)	0.296	6.8
	Blood	Ref		
Level of assistance	Unassisted	0.46 (0.37, 0.54)	<0.001	62.2
	Assisted	Ref		

### Willingness to trade-off for a more preferred HIVST attribute

To evaluate how much participants were willing to trade-off between attributes, marginal rates of substitution were calculated using the coefficients from the main effects model. Participants were willing to sacrifice 9.2 times the utility/benefit of preferred sample type, 5.1 times the utility of preferred delivery channel, and 3.3 times the utility of preferred distribution model in order to obtain unassisted HIVST (Table 5).

**Table 5:** Trade-offs between HIVST service attributes among AGYW who participated in the DCE.

Preferred attribute level gained	Attribute level sacrificed	β-preferred attribute (95% CI)	β-sacrificed attribute (95% CI)	Trade-off
Unassisted testing	Oral swab	0.46 (0.37, 0.54)	0.05 (-0.04, 0.13)	9.2
Unassisted testing	Primary delivery	0.46 (0.37, 0.54)	0.09 (0.00, 0.17)	5.1
Unassisted testing	Facility-based distribution	0.46 (0.37, 0.54)	0.14 (0.06, 0.23)	3.3
Facility-based distribution	Oral swab	0.14 (0.06, 0.23)	0.05 (-0.04, 0.13)	2.8
Primary delivery	Oral swab	0.09 (0.00, 0.17)	0.05 (-0.04, 0.13)	1.8
Facility-based distribution	Primary delivery	0.14 (0.06, 0.23)	0.09 (0.00, 0.17)	1.6

### Assessment of interaction between attributes (final model)

Only one interaction term between the delivery channel and the level of assistance was statistically significant in the final model (β = 0.66 (95% CI: 0.07, 1.24) (Table 6), indicating that utility/benefit associated with the delivery channel varied according to whether HIVST was assisted or unassisted. When testing was assisted, there was no significant preference for primary over secondary delivery (β = 0.14 (95% CI: -0.56, 0.06)). In contrast, when testing was unassisted, primary delivery was significantly preferred over secondary delivery (β = 0.41 (95% CI: 0.11, 0.71)). Similarly, under secondary delivery, there was no significant preference between unassisted and assisted testing (β = 0.13 (95% CI: -0.18, 0.43)). Whereas under primary delivery, unassisted testing was strongly preferred over assisted testing (β = 0.78 (95% CI: 0.48, 1.08)).

**Table 6:** A mixed logit model examining the interaction between HIVST delivery channel and level of assistance.

Attribute	Attribute level	Coefficient (95% CI)	P value
Distribution model	Facility-based	0.14 (0.06, 0.23)	0.001
	Community-based	Ref	
Delivery channel	Primary delivery	-0.25 (-0.56, 0.06)	0.118
	Secondary delivery	Ref	
Sample	Oral swab	0.05 (-0.04, 0.13)	0.292
	Blood	Ref	
Level of assistance	Unassisted testing	0.13 (-0.18, 0.43)	<0.416
	Assisted testing	Ref	
Delivery channel X Level of assistance	Primary delivery X unassisted testing	0.66 (0.07, 1.24)	0.028
When testing is assisted	Primary delivery	-0.25 (-0.56, 0.06)	0.118
	Secondary delivery	Ref	
When testing is unassisted	Primary delivery	0.41 (0.11, 0.71)	0.007
	Secondary delivery	Ref	
When delivery is secondary	Unassisted testing	0.13 (-0.18, 0.43)	0.415
	Assisted testing	Ref	
When delivery is primary	Unassisted testing	0.78 (0.48, 1.08)	<0.001
	Assisted testing	Ref	

### Predicted utility and uptake of HIVST service profiles

The predicted utility and uptake were based on the interaction model. The model profiles that paired primary delivery channel and unassisted testing were highly preferred. The model profile that had facility-based distribution model, primary delivery channel, oral test kit and unassisted HIVST had the highest predicted utility 0.72 (95% CI: 0.56, 0.89) and highest predicted uptake of HIVST 91.3 (95% CI: 90.0, 92.6). The top four ranking model profiles utilized primary delivery channel coupled with unassisted testing, and has predicted uptake rates ≥89% (Table 7).

**Table 7:** The predicted utility and uptake of HIVST delivered through the 16 selected model profiles among AGYW.

Rank	Distribution model	Delivery channel	Sample type	Level of assistance	Profile utility*	Predicted uptake (%) ^$^
1	Facility-based	Primary	Oral swab	Unassisted	0.72 (0.56, 0.89)	91.3 (90.0, 92.6)
2	Facility-based	Primary	Blood	Unassisted	0.68 (0.54, 0.82)	90.9 (89.7, 92.1)
3	Community-based	Primary	Oral swab	Unassisted	0.58 (0.44, 0.72)	90.1 (88.8, 91.4)
4	Community-based	Primary	Blood	Unassisted	0.54 (0.41, 0.66)	89.7 (88.5, 90.8)
5	Facility-based	Secondary	Oral swab	Unassisted	0.31 (-0.01, 0.64)	87.4 (83.8, 91.0)
6	Facility-based	Secondary	Blood	Unassisted	0.27 (-0.05, 0.58)	86.9 (83.3, 90.5)
7	Facility-based	Secondary	Oral swab	Assisted	0.19 (0.06, 0.31)	86.0 (84.5, 87.5)
8	Community-based	Secondary	oral	Unassisted	0.17 (-0.14, 0.49)	85.8 (81.9, 89.6)
9	Facility-based	Secondary	Blood	Assisted	0.14 (0.06, 0.23)	85.4 (84.4, 86.5)
10	Community-based	Secondary	Blood	Unassisted	0.13 (-0.18, 0.43)	85.2 (81.4, 89.1)
11	Community-based	Secondary	Oral swab	Assisted	0.05 (-0.04, 0.13)	84.2 (83.1, 85.1)
12	Community-based	Secondary	Blood	Assisted	0.00 (Reference)	83.6
13	Community-based	Secondary	Oral swab	Assisted	-0.06 (-0.39, 0.27)	82.7 (78.0, 87.4)
14	Facility-based	Primary	Blood	Assisted	-0.10 (-0.42, 0.21)	82.1 (77.4, 86.8)
15	Community-based	Primary	Oral swab	Assisted	-0.20 (-0.52, 0.12)	80.6 (75.6, 85.6)
16	Community-based	Primary	Blood	Assisted	-0.25 (-0.56, 0.06)	79.9 (74.9, 84.8)

*Profile utility represents the utility/benefit derived from each profile of HIVST service attributes relative to the reference levels (community-based distribution, secondary delivery channel, blood sample and assisted testing).

$ The predicted uptake represents the model-predicted probability of choosing the specified HIVST profile rather than opting out (choosing none of the profile).

### Sensitivity analysis

Only 4 out 340 participants (1.2%) exhibited straight-lining behavior.

Leave-one-choice-set-out sensitivity analysis showed that the direction and magnitude of the estimated coefficients remained stable across all models. The preference for unassisted HIV self-testing remained strong and statistically significant in all models, while facility-based distribution and primary delivery retained positive effects (S4 File).

## Discussion

This DCE explored HIVST service preferences among AGYW at a higher risk of HIV in urban Uganda to identify preferred service delivery models. Findings show a strong preference for models with unassisted HIVST, facility-based distribution, and primary-level delivery channels, while the type of test kit (oral vs. blood) had no significant influence on choice of the alternative tasks. These results provide actionable evidence to guide HIVST models that are responsive to the needs and realities of AGYW.

The level of assistance emerged as the most influential attribute, contributing over 60% of the relative importance in choice decisions. For AGYW, the ability to self-test without assistance was by far the most important factor in deciding whether to use the HIVST service model. It was three times more important than the source of the kit, five times more important than the delivery channel, and nine times more important than the type of test kit. This indicates that even if kit type or distribution method is not ideal, AGYW are still more likely to use HIVST if they can do it without assistance. Moreover, we found that the level of assistance influenced the effect of delivery channel on HIVST preferences. The preference for primary delivery was significantly stronger when HIVST was unassisted, but when HIVST is assisted, the mode of delivery appeared to be less influential. This finding aligns with global evidence that autonomy and privacy are key facilitators of HIVST uptake [41,42]. Importantly, primary delivery of the HIVST kits may reduce reliance on intermediaries and enhance privacy. Unassisted testing offers control over the testing process while minimizing stigma from health workers, peers, or partners – barriers that AGYW are particularly vulnerable to [43,44]. It also reduces practical obstacles such as scheduling, travel, and waiting times, while allowing immediate testing at moments of perceived risk. HIVST models targeting AGYW should therefore, prioritize unassisted testing to maximize acceptability and uptake.

The distribution model attribute ranked second in relative importance with facility-based distribution preferred over community-based distribution. This finding suggests that facility-based distribution should remain an important option in HIVST service models for AGYW. However, our DCE did not distinguish between the different types or levels of health facilities, therefore, we could not identify which specific facility-based distribution point would be most preferred. Previous studies have similarly reported that the location and mode of HIVST distribution influence user preferences [19,45], although preferences vary across populations and settings. Nonetheless, these findings highlight the importance of considering the local context and preferences of the target population when selecting distribution points for HIVST.

In the main-effects analysis, delivering HIVST kits through primary delivery channels provided greater utility than secondary distribution, however, the interaction analysis showed that this preference depends on the level of assistance. Primary delivery was preferred over secondary delivery when testing was unassisted but no difference was observed between delivery channels when testing was assisted. This pattern was also reflected in the profile simulations, where profiles combining primary delivery with unassisted testing had the highest utilities. Evidence from Zimbabwe and Malawi similarly shows that youth prefer collecting kits from health facilities or pharmacies because of trust, confidentiality, and independence [45]. Moreover, previous studies have identified privacy, confidentiality, autonomy and concerns about disclosure as important factors that may influence how HIVST services are accessed and delivered [46]. Although these factors were not directly assessed in our DCE, they provide useful context to understanding how delivery approaches may be perceived. Our findings suggest that HIVST service models for AGYW may be more attractive when primary delivery is paired with unassisted testing.

The sample type had relatively minor role in HIVST preferences in our study, with no clear preference between oral and blood-based tests. A recent systematic review of DCEs on HIV testing preferences found considerable heterogeneity in preference of testing modality across populations [17]. Similar studies among young people have reported mixed preferences for oral and blood-based HIVST [19,47]. In our study, the comparative small influence of sample type suggests that AGYW may place greater weight on how HIVST service is accessed rather and undertaken than on specimen used for testing. From a program perspective, this finding supports flexibility in offering oral- or blood-based HIVST kits where feasible, while prioritizing service delivery configurations that strongly shape preference among AGYW.

### Limitations

This study employed a DCE to elicit stated preferences using hypothetical scenarios, which may not fully reflect actual behavior when AGYW are offered HIVST options. This could result in either over or under-estimation of preferences. However, the sensitivity analysis was done to identify straight-lining behavior, of which just 1.2% (4/340) of the participants exhibited this behavior. This straight-lining behavior in our study was below the recommended threshold of <5% [48]. Furthermore, in the presentation of some attributes in our study as composite descriptions containing multiple delivery modalities to ensure contextual realism for participants, for example facility-based distribution (that encompasses, public and private hospitals, smaller clinics, pharmacies and drug shops) and community-based distribution that grouped several venues like VHT and drop-in centers, the findings do not differentiate between potential variations in preferences for the individual sub-options nested within the broader description. As HIVST becomes normalized, AGYW’s preferences may evolve over time; however, the cross-sectional design of this study did not allow assessment of such temporal changes. Although the attributes and levels were guided by Uganda Ministry of Health-recommended DSD models, the potential influence of cost, as an important determinant of HIVST uptake, was not assessed. Some data were collected retrospectively and are subject to recall bias, though this was minimized by restricting the recall period to six months (except for HIV testing history, which extended to 12 months). Convenient sampling – a non – probability technique, may have introduced selection bias by not giving every eligible participant an equal chance of participation. Similarly, the exclusion of participants who did not understand/speak English or Luganda could have introduced selection bias; however, Luganda is widely spoken in the study setting. Finally, the study focused on AGYW at a high-risk of acquiring HIV in urban settings, which may limit the generalizability of findings to rural or HIV lower-risk populations. Despite these limitations, the potential biases are unlikely to have substantially affected the validity of the findings.

## Conclusions

Among AGYW urban settings, HIVST service models offering facility-based distribution and combining primary delivery with unassisted testing were most preferred. These findings support considerations of such service configurations when designing HIVST programs for AGYW.

### Recommendations

From the findings, we recommend that Ministry of Health and HIV program implementing partners consider HIVST service models that combine primary delivery with unassisted testing while retaining facility-based distribution as an important access option for AGYW. Further studies are needed to determine the facility-based options most acceptable to AGYW and also examine the impact of other service attributes such as cost.

## Supporting information

S1 File HIV risk assessment tool. (PDF)

S2 File DCE questionnaire. (PDF)

S3 File Dataset. (DTA)

S4 File Table 8 (Sensitivity analysis). (PDF)
