# Supplementary material for: HIV self-testing preferences among adolescent girls and young women (AGYW) in urban settings in Uganda: A discrete choice experiment
Source: PLOS Glob Public Health. 2026 Sep 22;6(9):e0005431. doi: 10.1371/journal.pgph.0005431 (PMC13596813; doi:10.1371/journal.pgph.0005431)
Supplement: Supplementary file 1 [file pgph.0005431.s001.pdf]

Eligibility Assessment form

Study tittle: Barriers and facilitators of intention to use, and the preferred model for HIV Self-Testing among young women in the Kampala Metropolitan Area

Please complete this form before performing study procedures (after informed consent)

Participant ID: |___|.|___|.|___|

1. **What is the sex that was assigned to you at birth?**

1.|___| Female

2.|___| Male

2. What is your age in completed years?

|___|. |___|

Is the age in completed years between 15-24 years?

1.|___| Yes

2.|___| No

3. Have you stayed in the study district for more than 6 months?

1.|___| Yes

2.|___| No

4. **HIV risk assessment**

Is the HIV risk assessment score ≥2 including score of "1" from sexual activity?

1.|___| Yes

2.|___| No

5. Have provided written informed consent?

1.|___| Yes

2.|___| No

Does the participant fulfill the eligibility criteria?

1.|___| Yes

2.|___| No

If "Yes", continue with other study procedures

If "No", stop other study procedures

Completed by

____________________	____________________	____________________
**Name**	**Signature**	**Date**
