# Supplementary material for: HIV self-testing preferences among adolescent girls and young women (AGYW) in urban settings in Uganda: A discrete choice experiment
Source: PLOS Glob Public Health. 2026 Sep 22;6(9):e0005431. doi: 10.1371/journal.pgph.0005431 (PMC13596813; doi:10.1371/journal.pgph.0005431)
Supplement: Supplementary file 2 [file pgph.0005431.s002.pdf]

DCE questionnaire

Choice set 1

**Attribute**	**Option A**	**Option B**
Distribution model	Community-based model 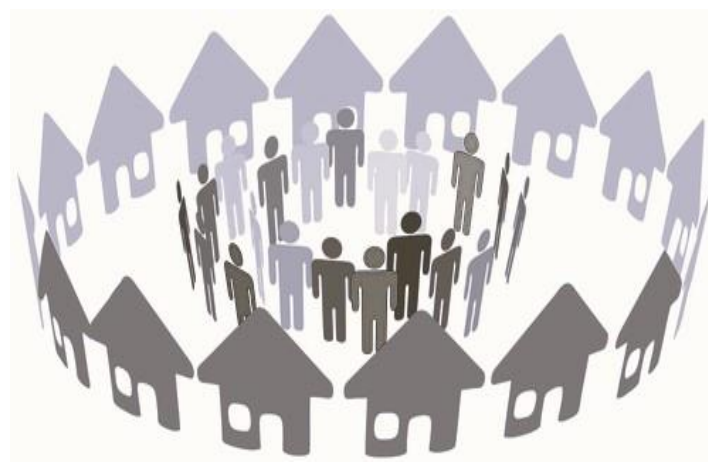	Facility-based model 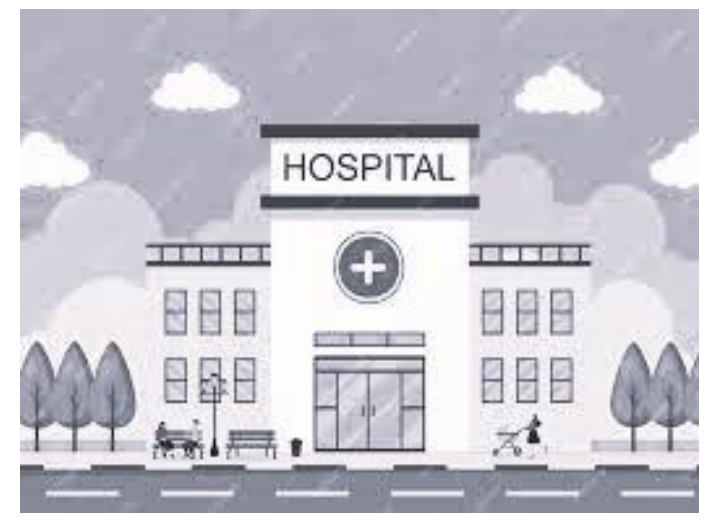
Delivery channel	Secondary delivery source 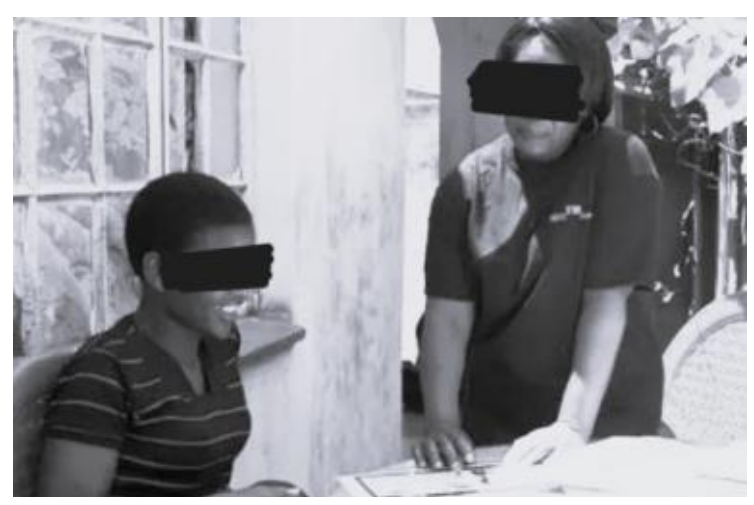	Primary delivery source 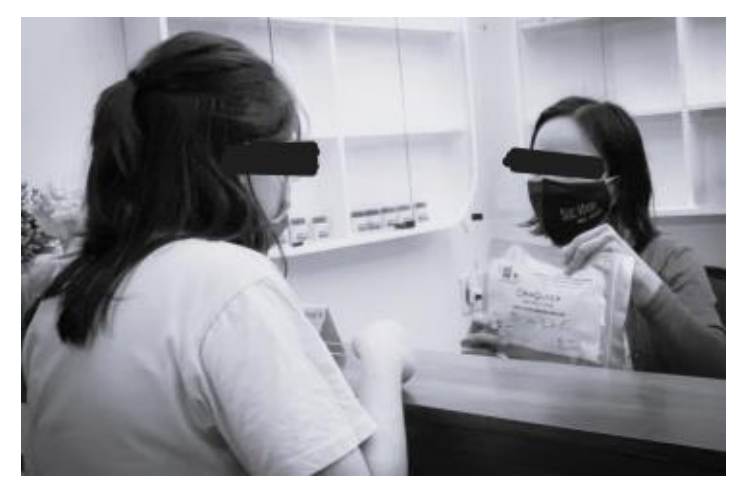
Sample	Oral swab 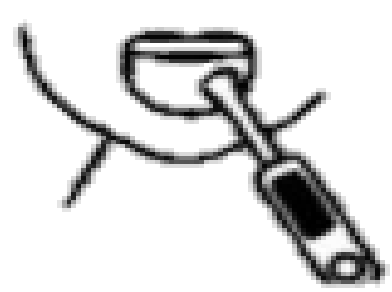	Blood sample 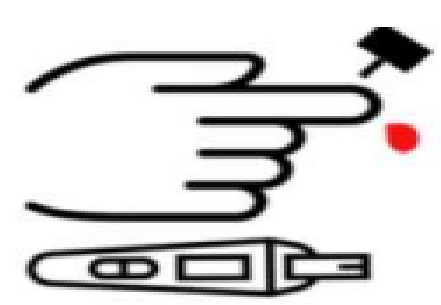
Test procedure	Unassisted 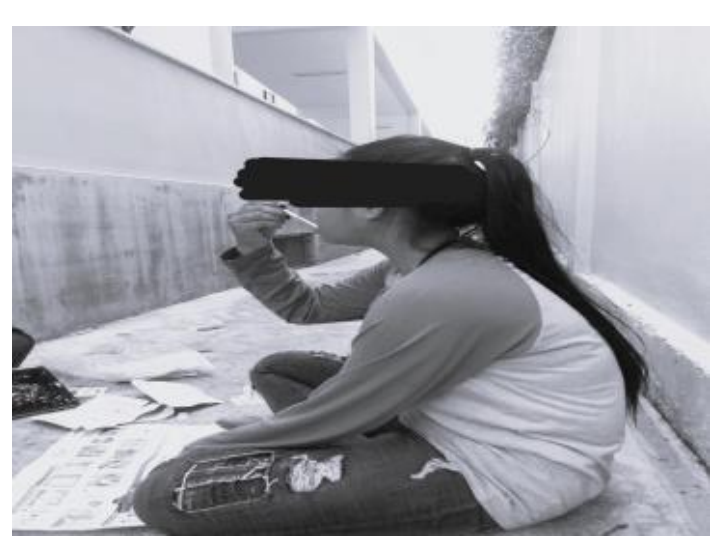	Assisted 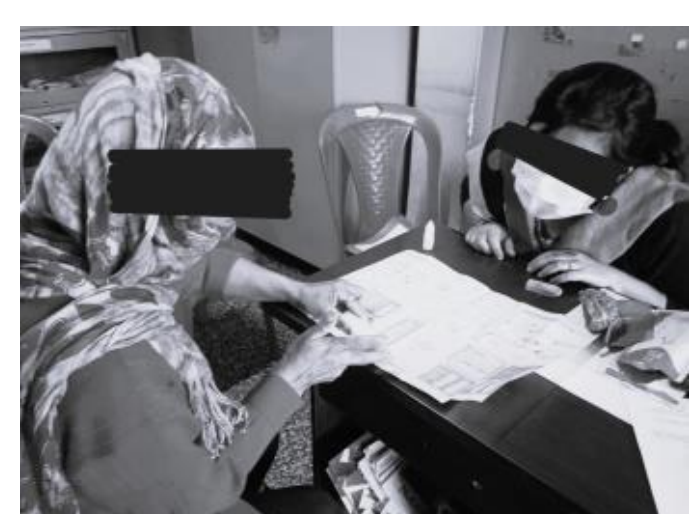
Participant choice	1. |___| Option A<br>2. |___|Option B<br>3. |___| None/Opt-out	

Choice set 2

**Attribute**	**Option A**	**Option B**
Distribution model	Facility-based model 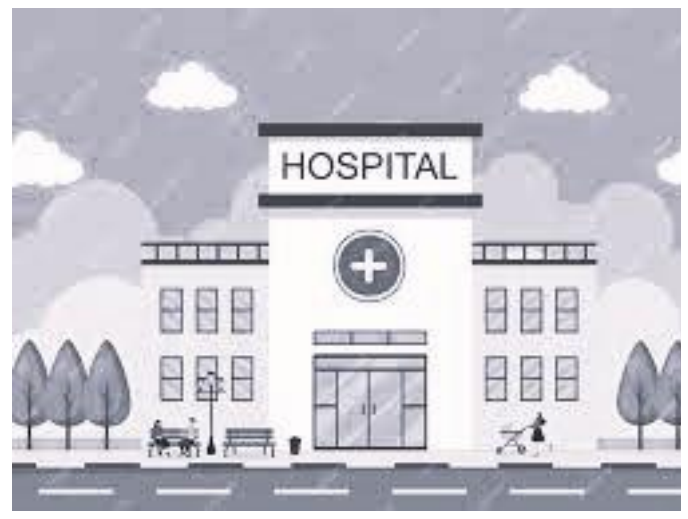	Community-based model 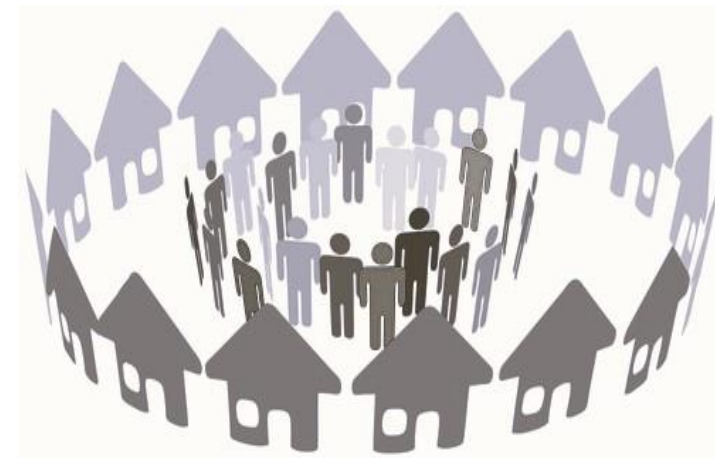
Delivery channel	Secondary delivery source 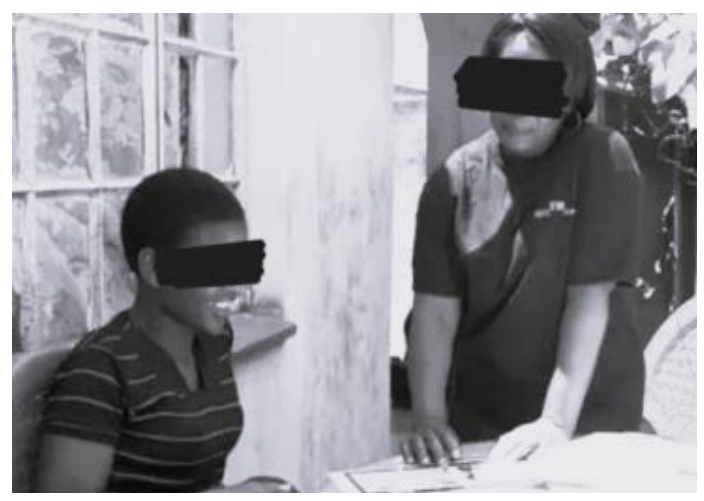	Primary delivery source 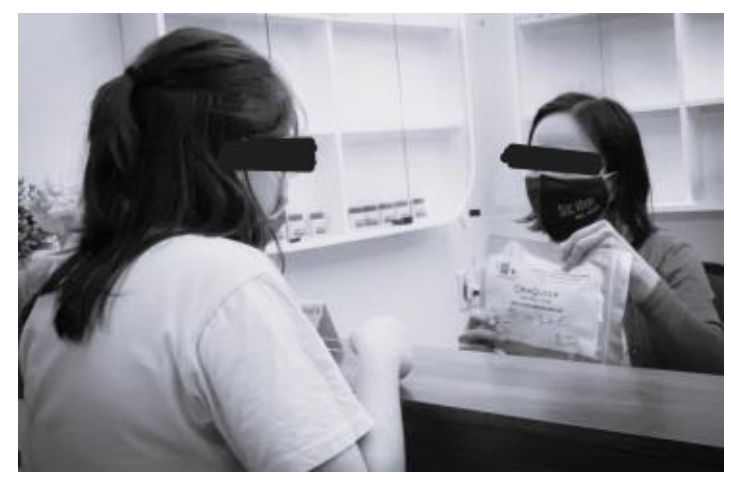
Sample	Oral swab 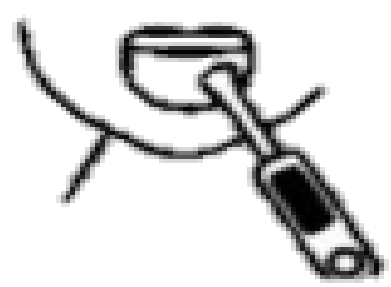	Blood sample 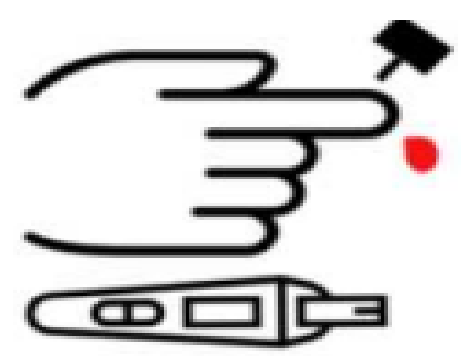
Test procedure	Assisted 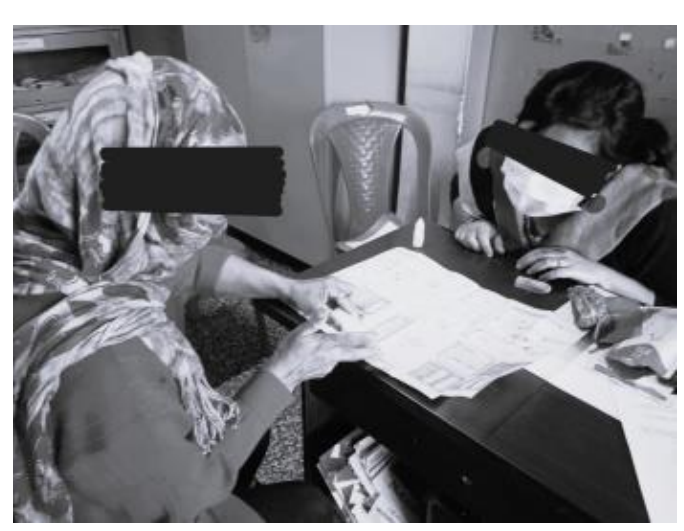	Unassisted 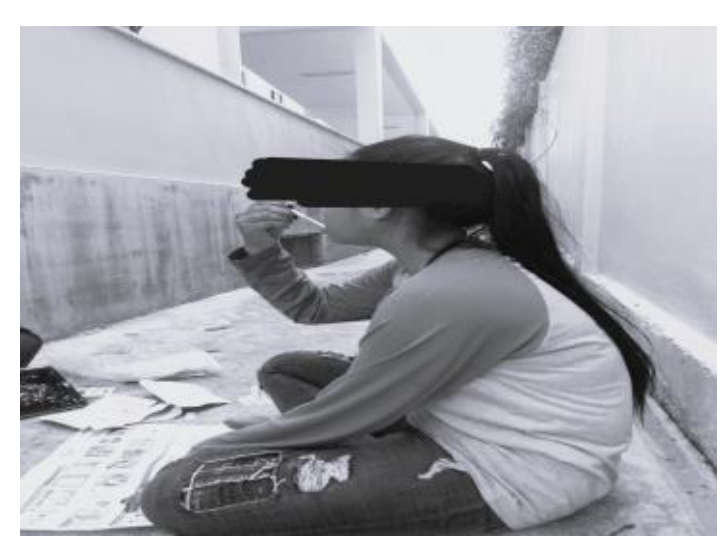
Participant choice	1. |___| Option A<br>2. |___|Option B<br>3. |___| None/Opt-out	

Choice set 3

**Attribute**	**Option A**	**Option B**
Distribution model	Facility-based model 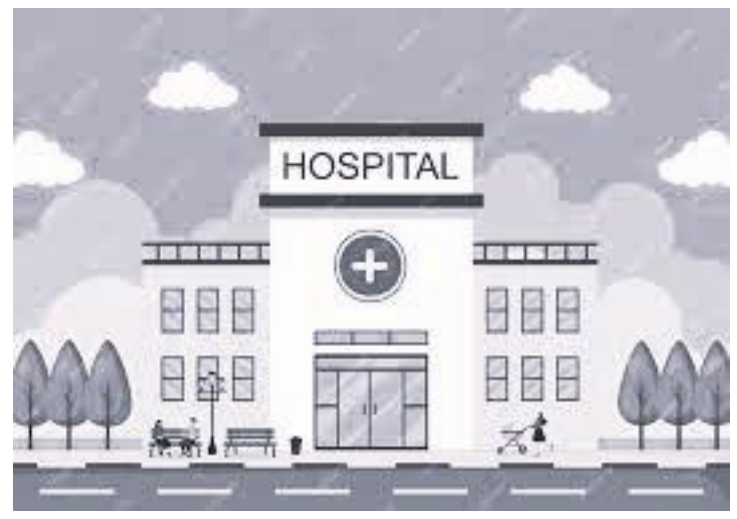	Community-based model 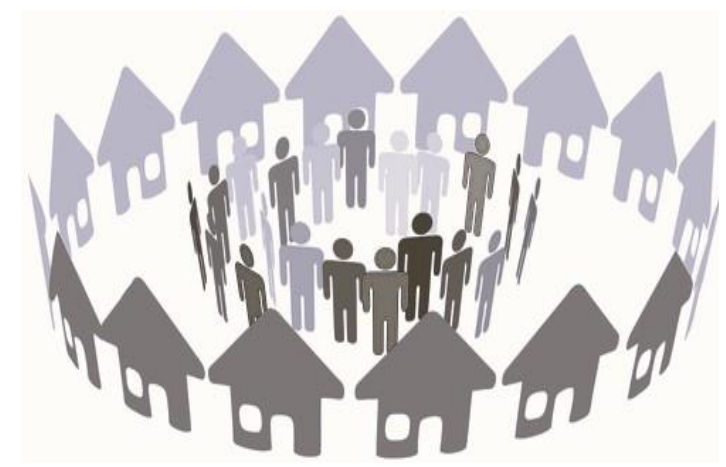
Delivery channel	Primary delivery source 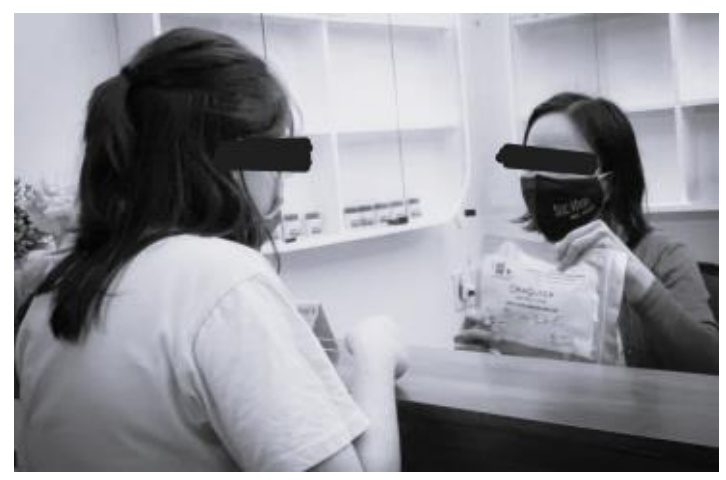	Secondary delivery source 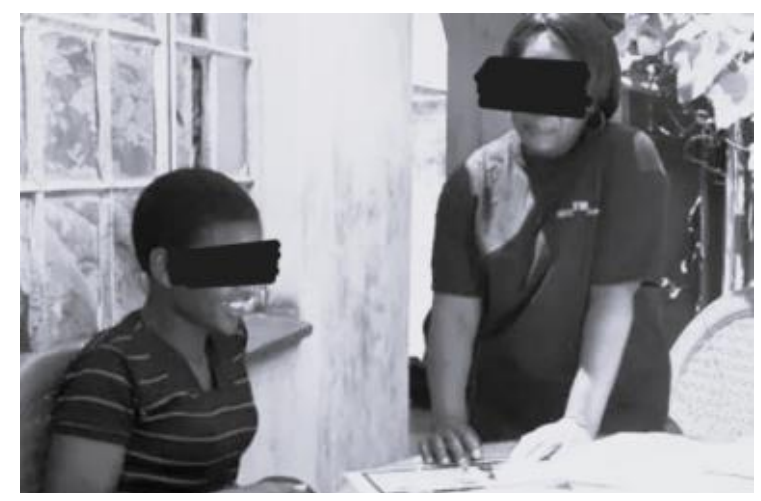
Sample	Oral swab 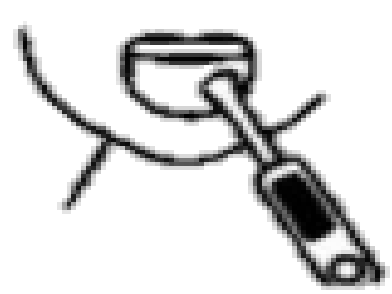	Blood sample 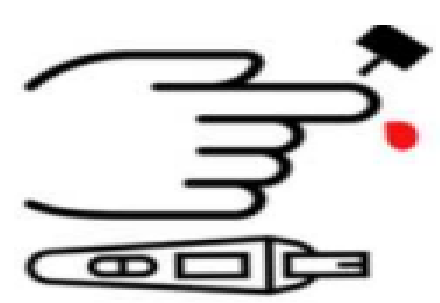
Test procedure	Unassisted 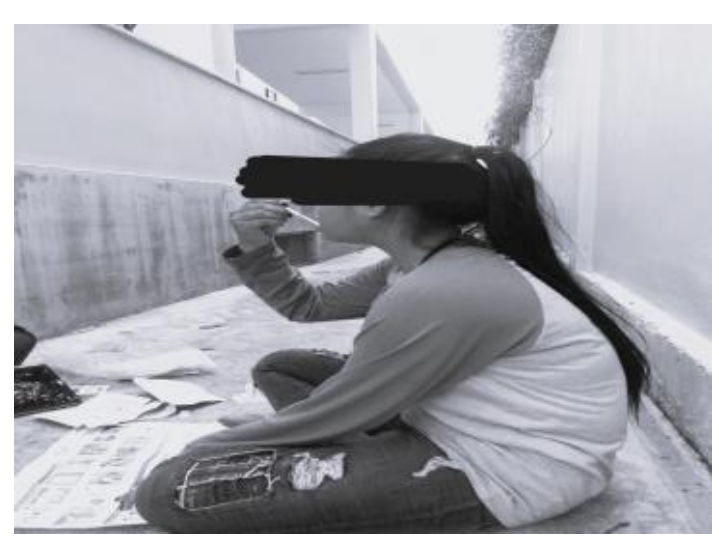	Assisted 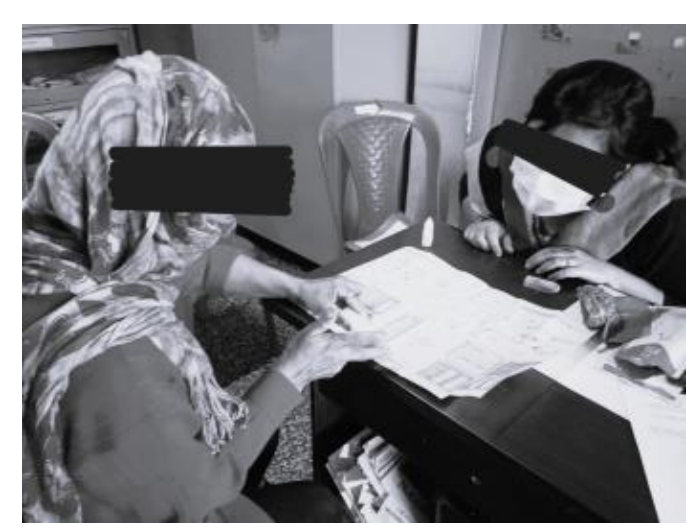
Participant choice	1. |___| Option A<br>2. |___|Option B<br>3. |___| None/Opt-out	

Choice set 4

Attribute	Option A	Option B
Distribution model	Facility-based model 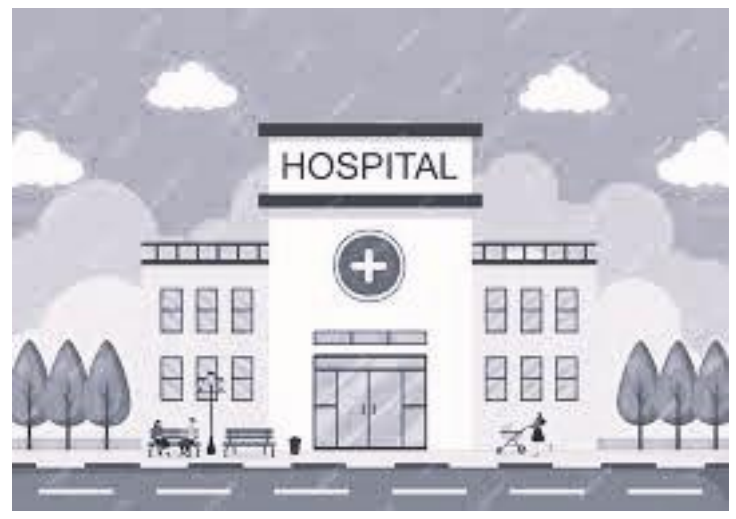	Community-based model 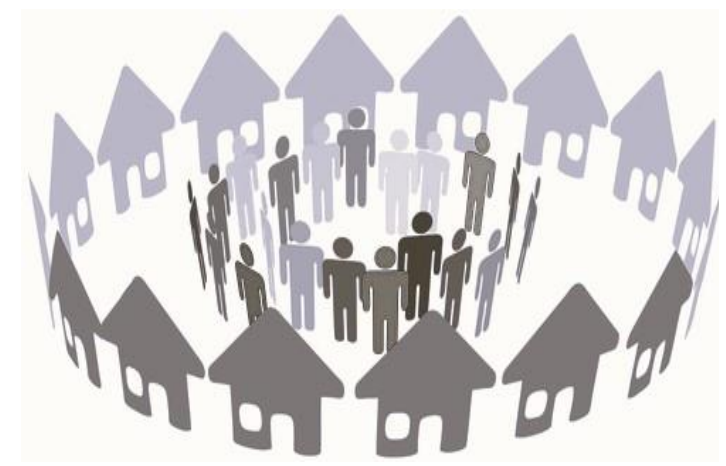
Delivery channel	Secondary delivery source 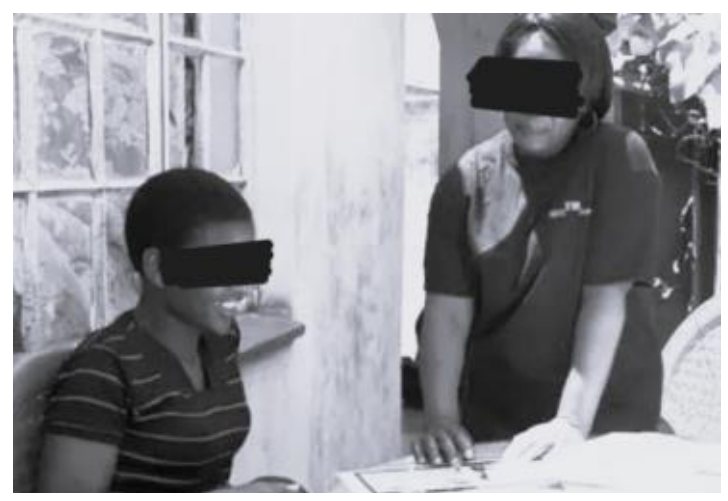	Primary delivery source 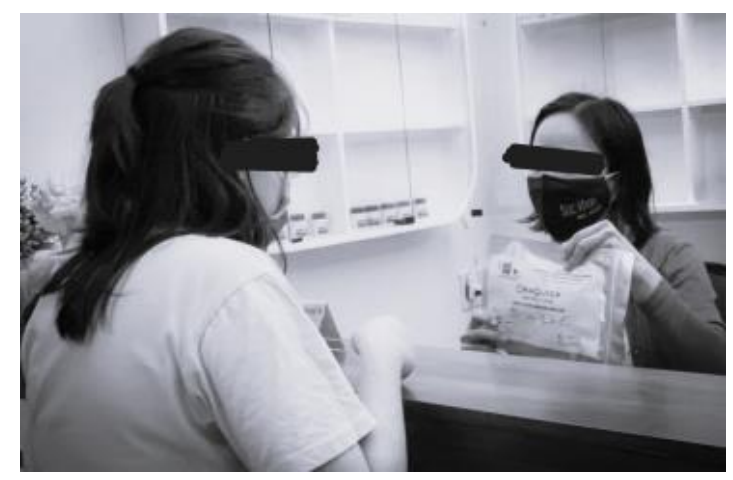
Sample	Blood sample 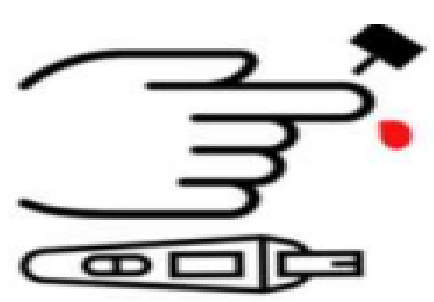	Oral swab 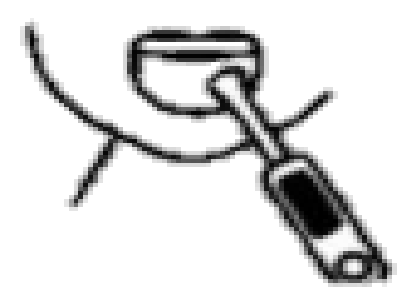
Test procedure	Unassisted 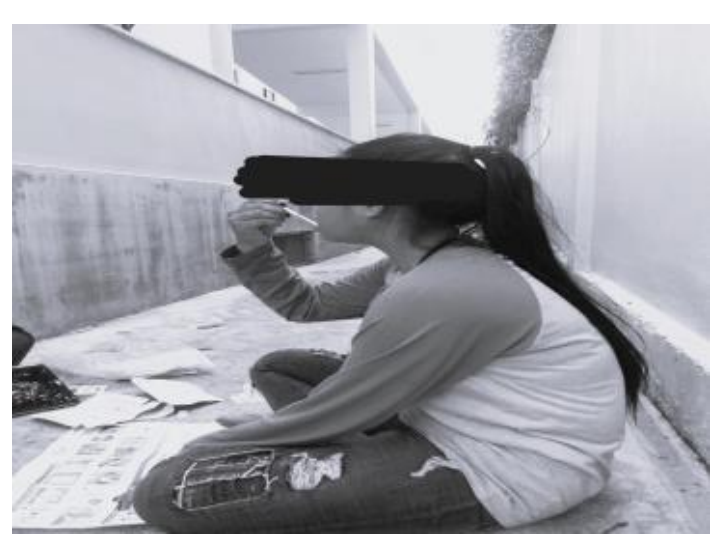	Assisted 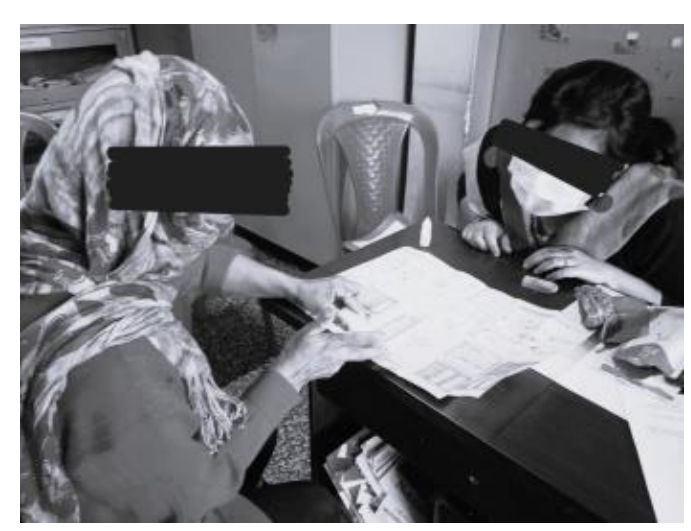
Participant choice	1. |___| Option A<br>2. |___|Option B<br>3. |___| None/Opt-out	

Choice set 5

**Attribute**	**Option A**	**Option B**
Distribution model	Facility-based model<br>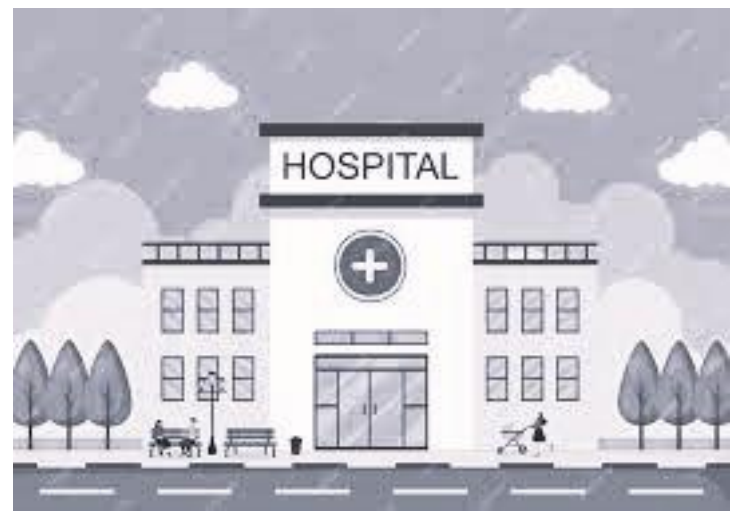	Community based model<br>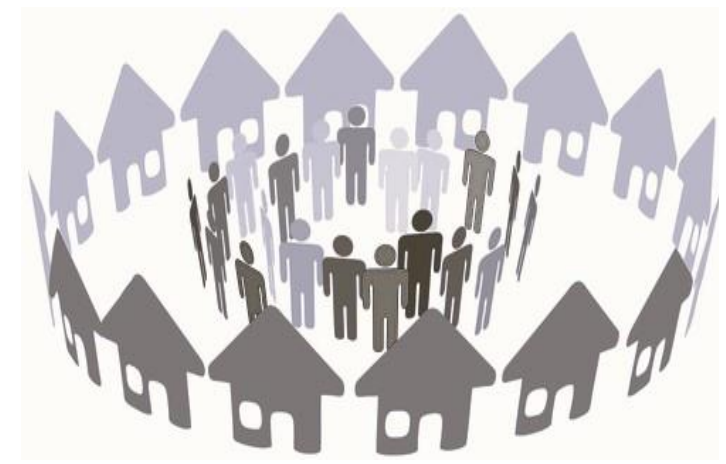
Delivery channel	Primary delivery source<br>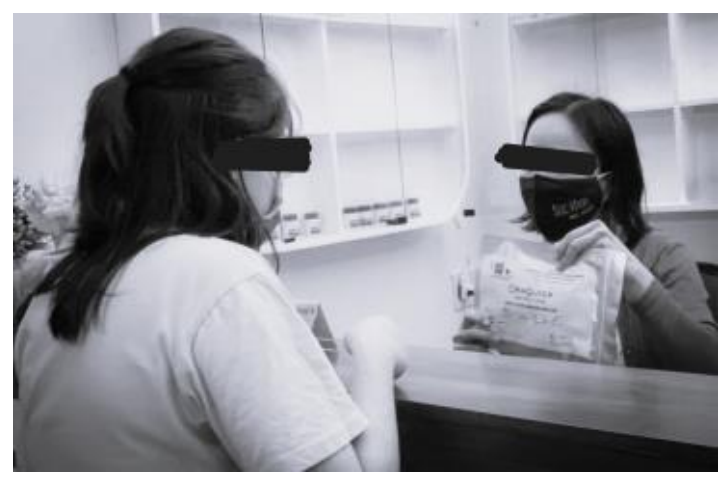	Secondary delivery source<br>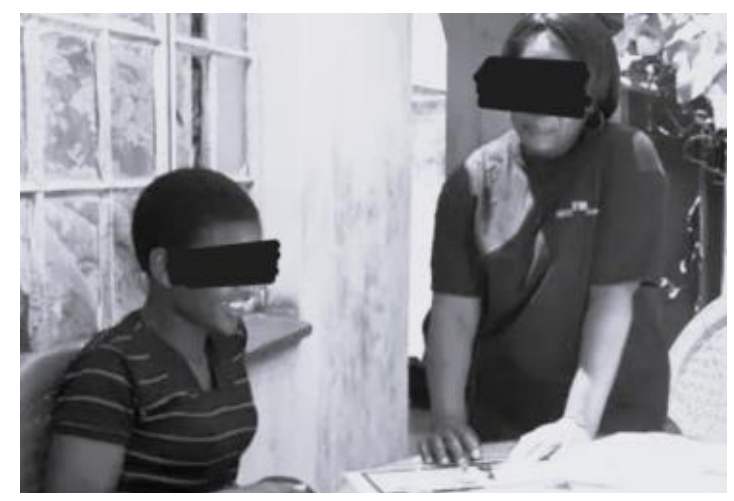
Sample	Oral swab<br>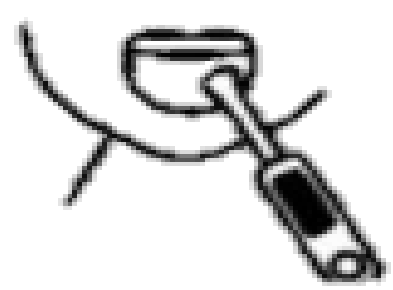	Blood sample<br>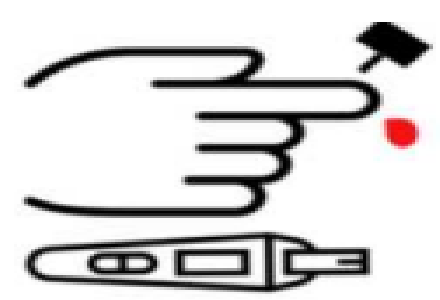
Test procedure	Assisted<br>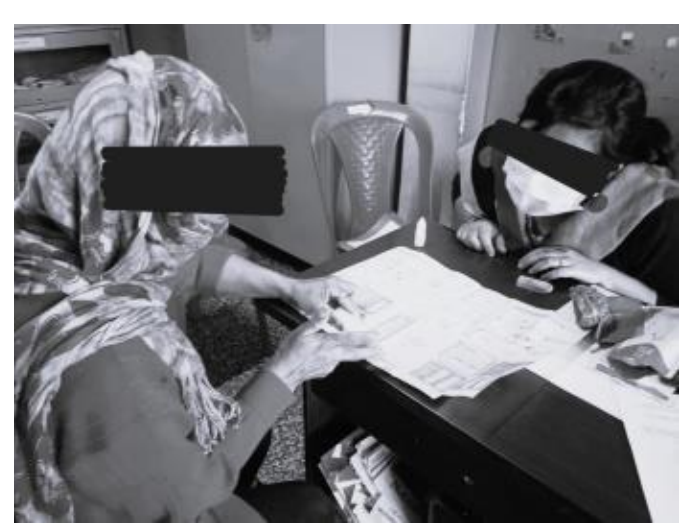	Unassisted<br>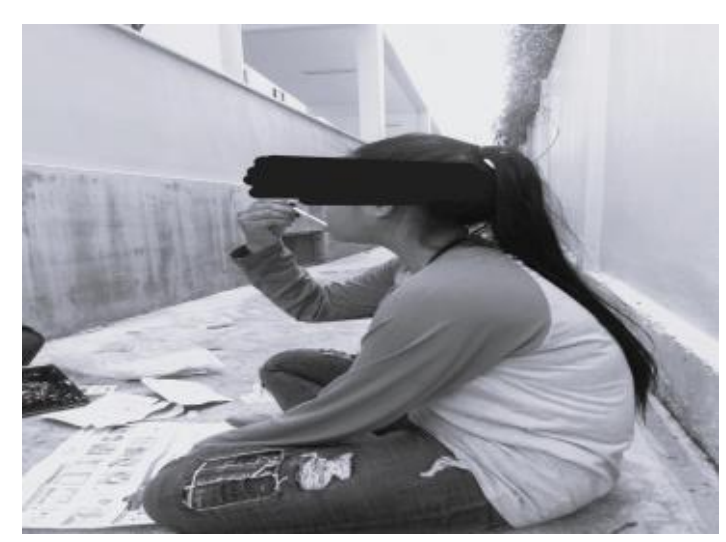
Participant choice	1. |___| Option A<br>2. |___|Option B<br>3. |___| None/Opt-out	

Choice set 6

Attribute	Option A	Option B
Distribution model	Facility-based model 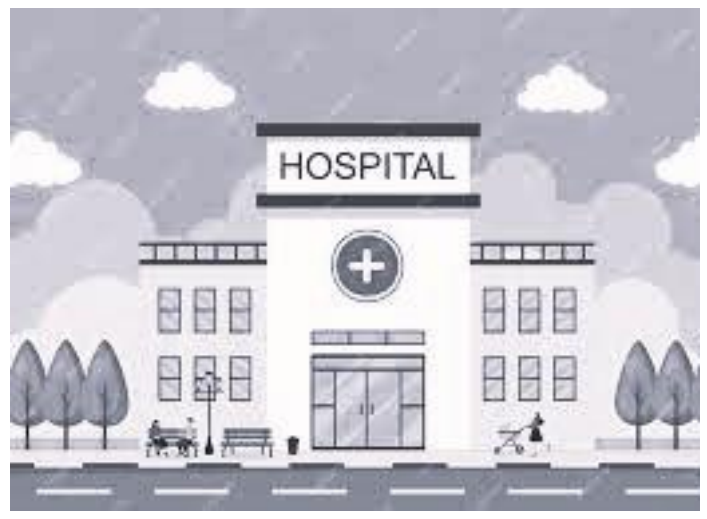	Community based model 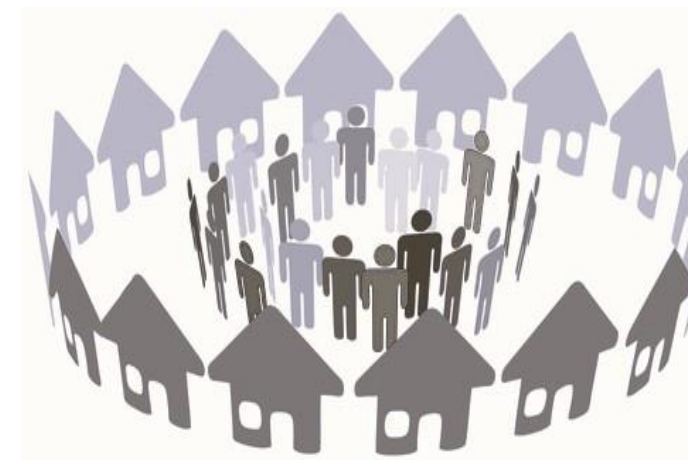
Delivery channel	Secondary delivery source 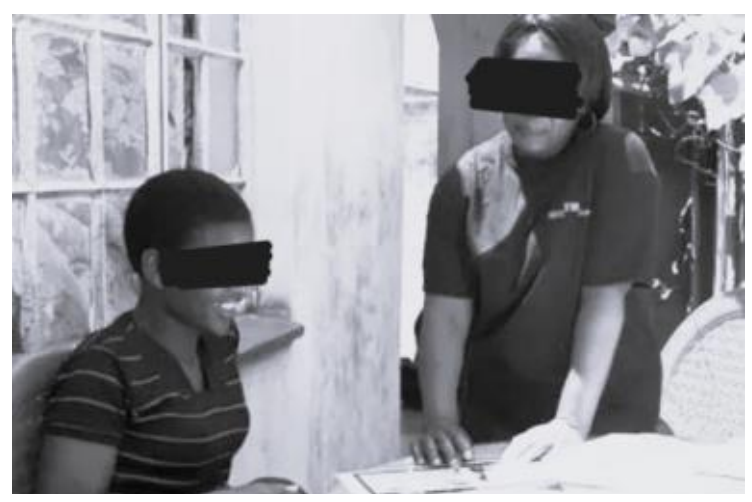	Primary delivery source 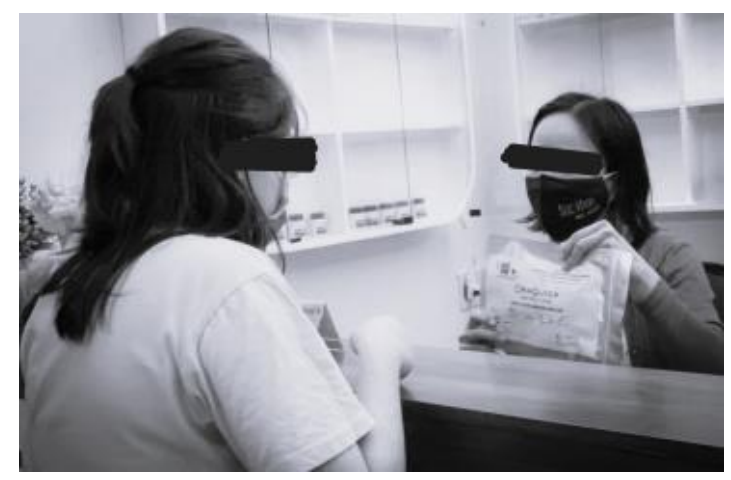
Sample	Blood sample 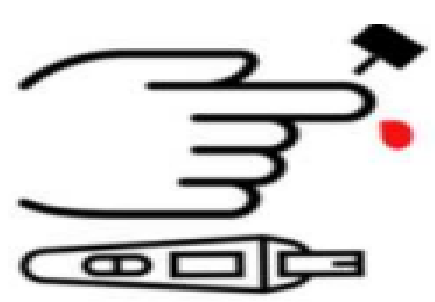	Oral swab 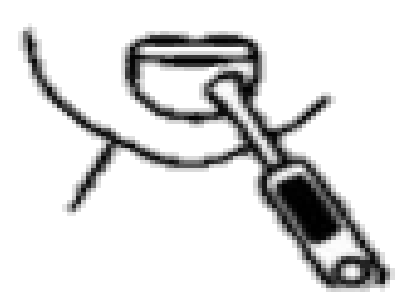
Test procedure	Assisted 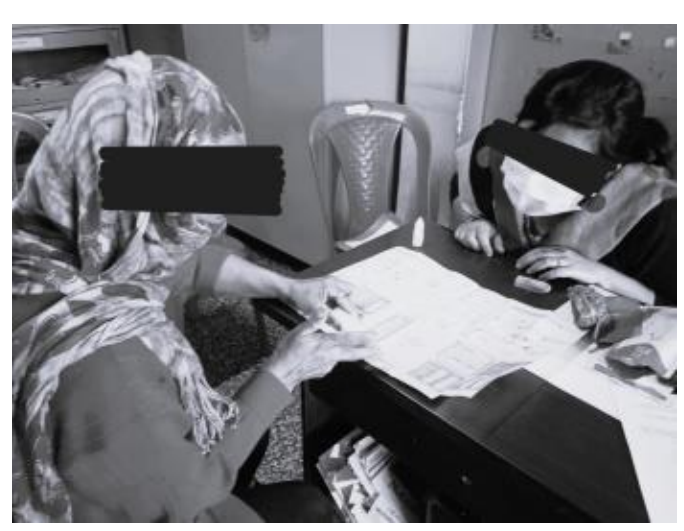	Unassisted 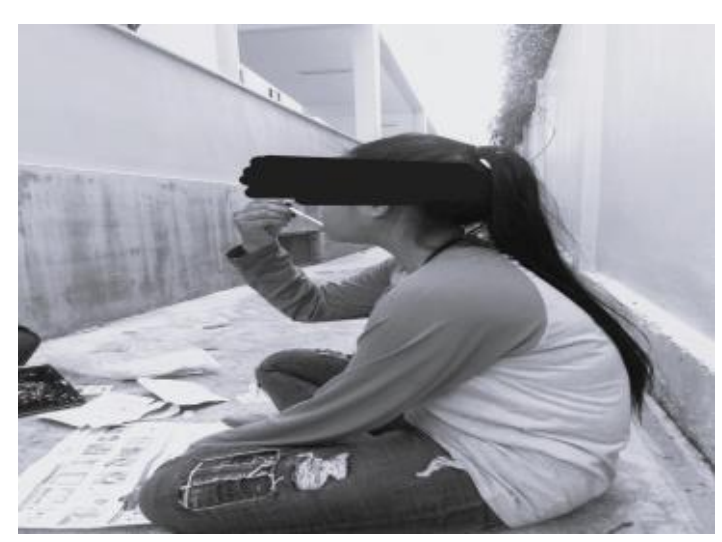
Participant choice	1. |___| Option A<br>2. |___|Option B<br>3. |___| None/Opt-out	

Choice set 7

**Attribute**	**Option A**	**Option B**
Distribution model	Community-based model 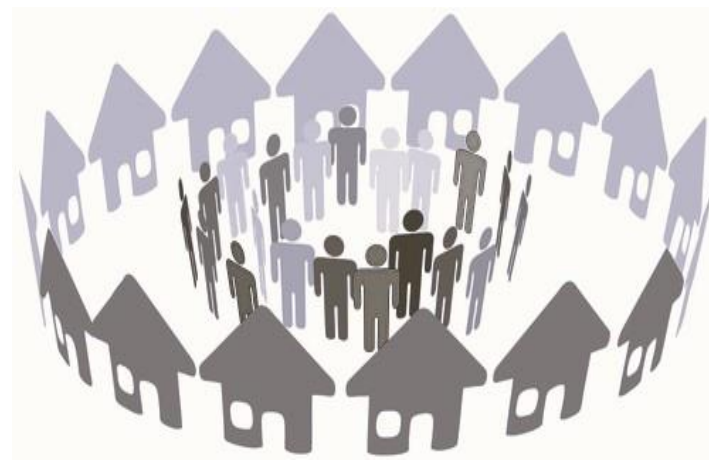	Facility-based model 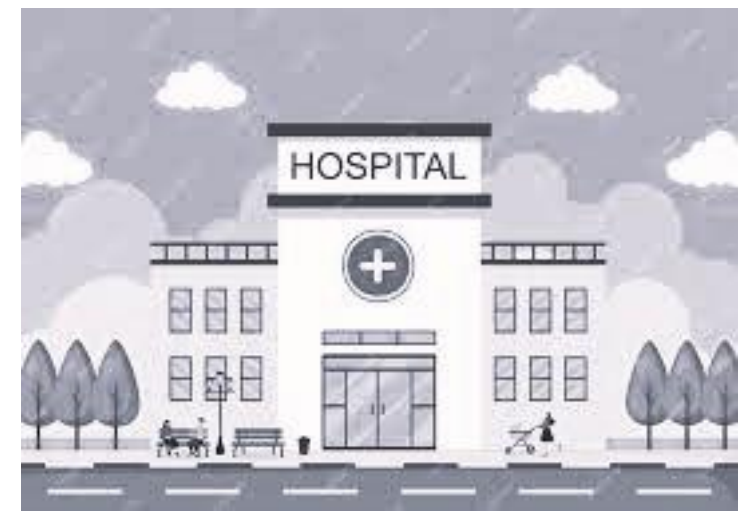
Delivery channel	Primary delivery source 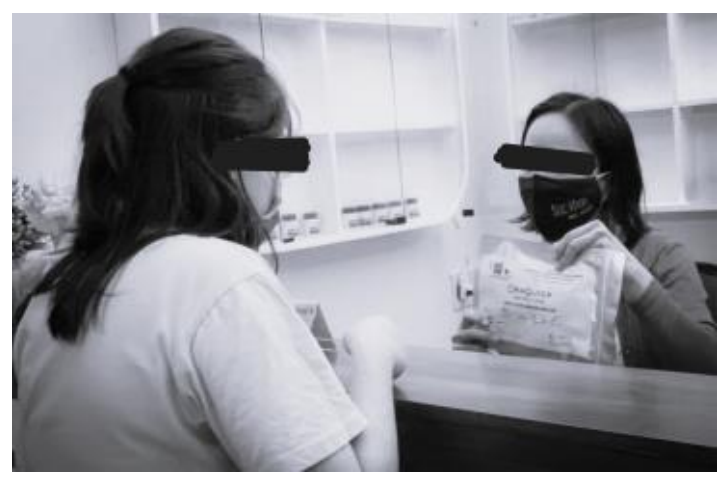	Secondary delivery source 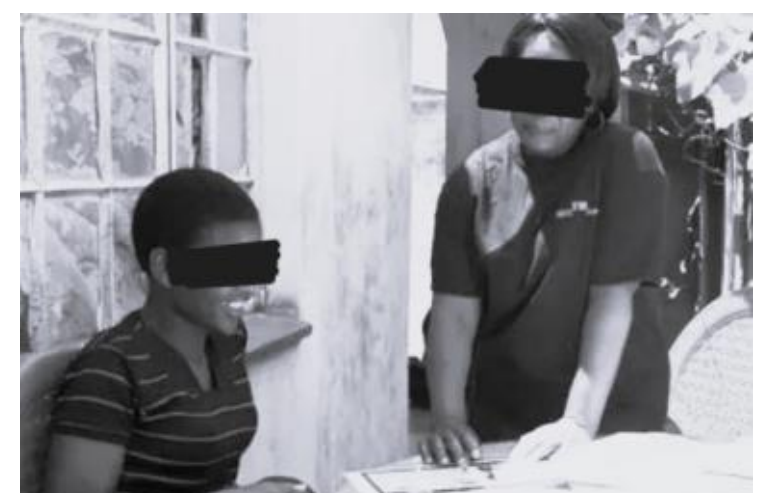
Sample	Blood sample 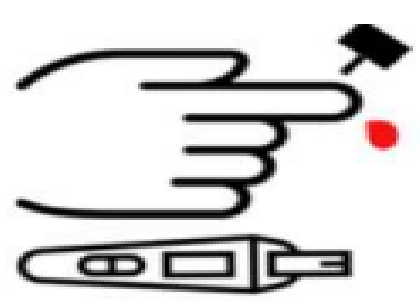	Oral swab 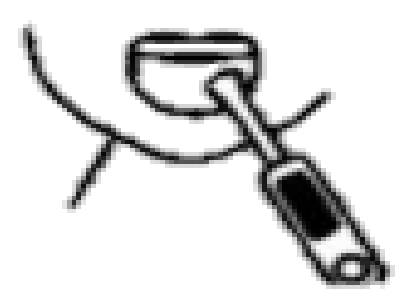
Test procedure	Assisted 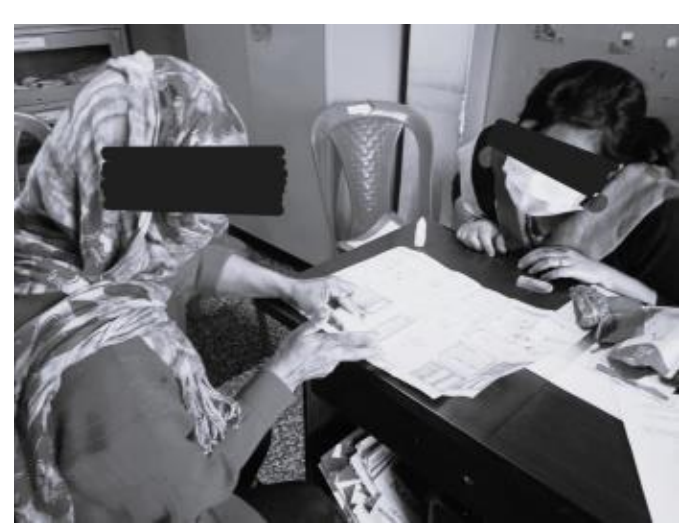	Unassisted 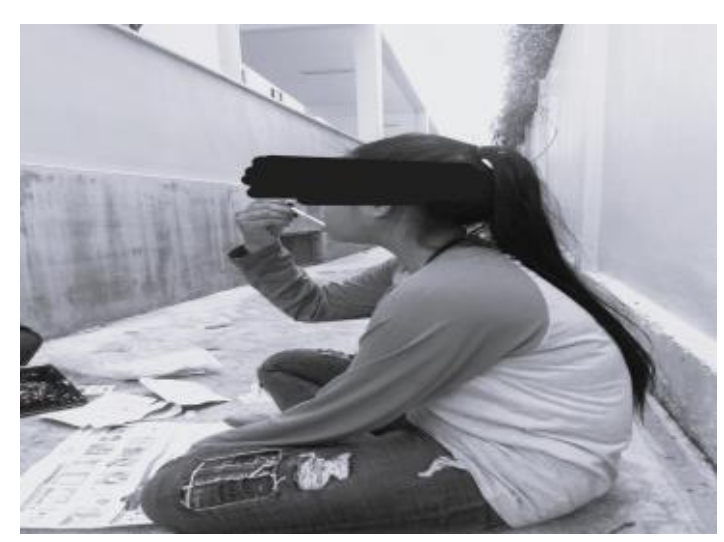
Participant choice	1. |___| Option A<br>2. |___|Option B<br>3. |___| None/Opt-out	

Choice set 8

Attribute	Option A	Option B
Distribution model	Community-based model 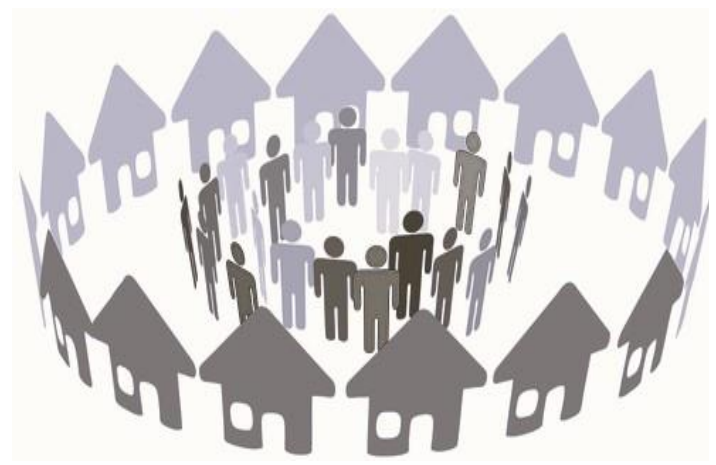	Facility-based model 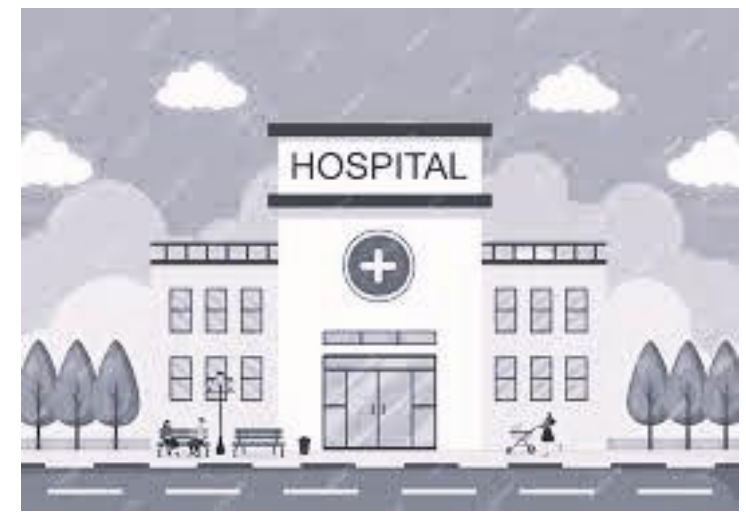
Delivery channel	Secondary delivery source 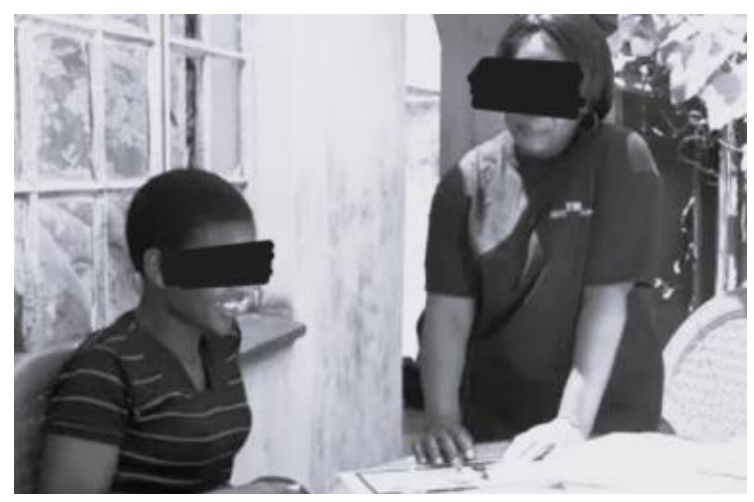	Primary delivery source 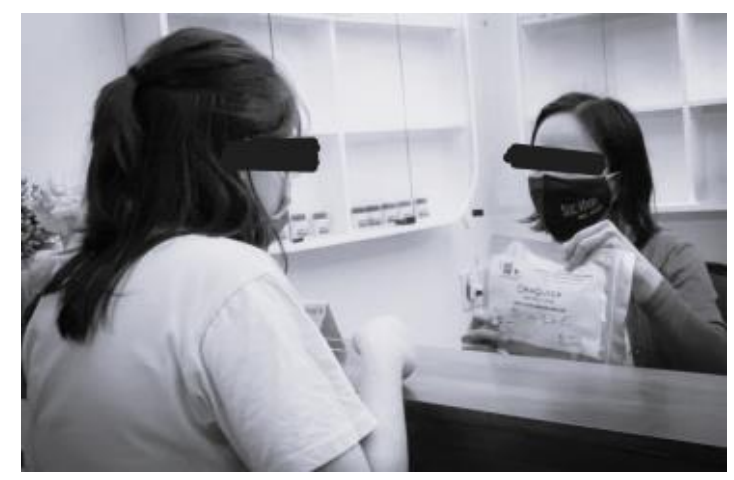
Sample	Oral swab 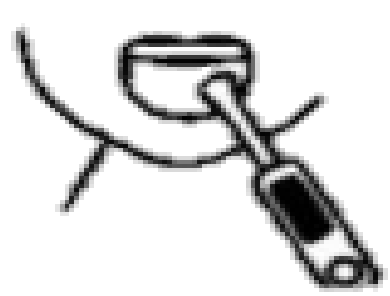	Blood sample 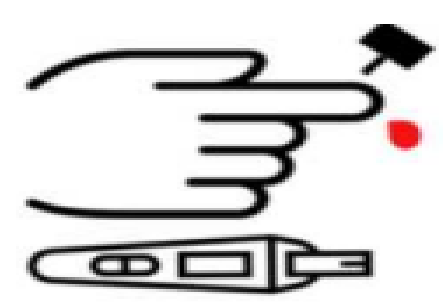
Test procedure	Assisted 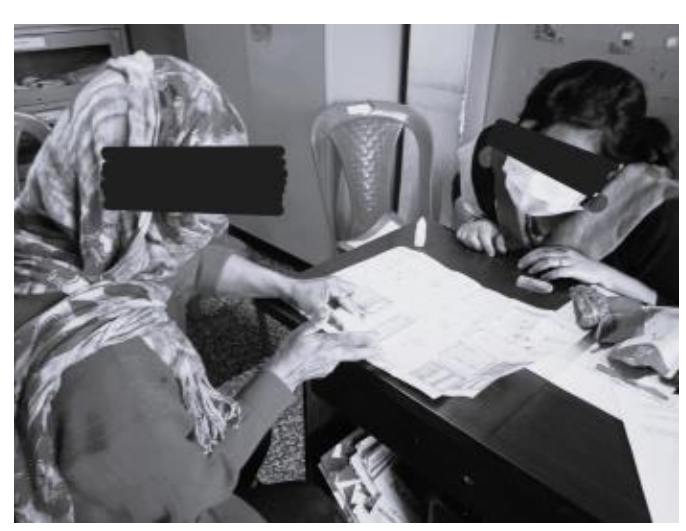	Unassisted 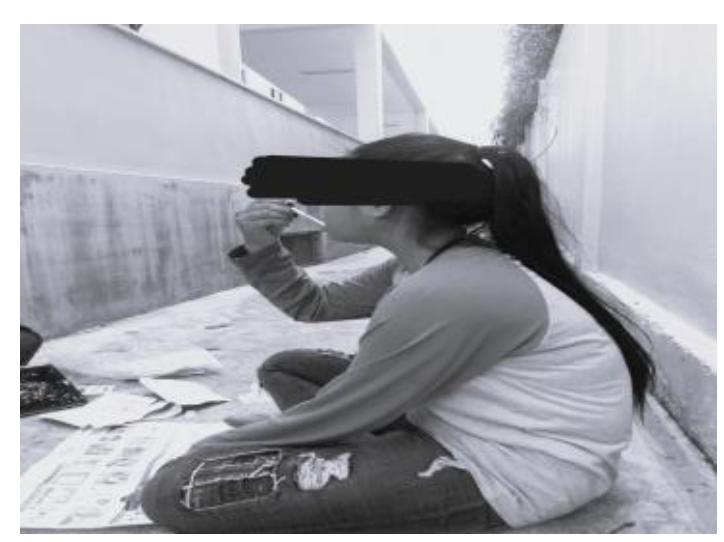
Participant choice	1. |___| Option A<br>2. |___|Option B<br>3. |___| None/Opt-out	

Thank you for your participation
