# Supplementary material for: HIV self-testing preferences among adolescent girls and young women (AGYW) in urban settings in Uganda: A discrete choice experiment
Source: PLOS Glob Public Health. 2026 Sep 22;6(9):e0005431. doi: 10.1371/journal.pgph.0005431 (PMC13596813; doi:10.1371/journal.pgph.0005431)
Supplement: Supplementary file 3 [file pgph.0005431.s004.pdf]

Sensitivity analysis

The leave-one-choice-set-out sensitivity analysis showed that the direction and magnitude of the estimated coefficients remained stable across all models. The preference for unassisted HIV self-testing remained strong and statistically significant in all models, while facility-based distribution and primary delivery retained positive effects.

Table 1: Sensitivity analysis – model estimates after excluding one choice set at a time

Model	Facility-based distribution β (95% CI)	Primary delivery β (95% CI)	Oral swab β (95% CI)	Unassisted testing β (95% CI)
Full model	0.14 (0.06, 0.23)	0.09 (0.00, 0.17)	0.05 (-0.04, 0.13)	0.46 (0.37, 0.54)
Excluding choice set 1	0.14 (0.05, 0.22)	0.08 (0.00, 0.17)	0.04 (-0.04, 0.13)	0.45 (0.37, 0.54)
Excluding choice set 2	0.15 (0.06, 0.23)	0.09 (0.01, 0.17)	0.05 (-0.04, 0.13)	0.46 (0.37, 0.54)
Excluding choice set 3	0.14 (0.06, 0.23)	0.09 (0.00, 0.17)	0.04 (-0.04, 0.13)	0.46 (0.37, 0.54)
Excluding choice set 4	0.14 (0.06, 0.23)	0.09 (0.00, 0.17)	0.05 (-0.04, 0.13)	0.46 (0.37, 0.54)
Excluding choice set 5	0.14 (0.06, 0.22)	0.09 (0.00, 0.17)	0.04 (-0.04, 0.13)	0.45 (0.37, 0.54)
Excluding choice set 6	0.15 (0.06, 0.23)	0.09 (0.01, 0.17)	0.05 (-0.04, 0.13)	0.46 (0.38, 0.55)
Excluding choice set 7	0.14 (0.06, 0.23)	0.09 (0.00, 0.17)	0.05 (-0.034, 0.13)	0.46 (0.37, 0.54)
Excluding choice set 8	0.14 (0.05, 0.22)	0.08 (0.00, 0.17)	0.04 (-0.04, 0.13)	0.45 (0.37, 0.53)
